# Supplementary material for: A comparative analysis of planarian genomes reveals regulatory conservation in the face of rapid structural divergence
Source: Nat Commun. 2024 Sep 19;15:8215. doi: 10.1038/s41467-024-52380-9 (PMC11410931; doi:10.1038/s41467-024-52380-9)
Supplement: Supplementary file 1 — Supplementary Information [file 41467_2024_52380_MOESM1_ESM.pdf]

A comparative analysis of planarian genomes reveals regulatory conservation in the face of rapid structural divergence.

Table of Contents

1 Genomic resources 2

1.1 Assembly quality assessment 2
1.2 Comparison to schMedS2 4
1.3 Phasing efficiency 5
1.4 Gene annotation benchmarking 6
1.5 Chimeric gene annotations 7

2 Regulatory element annotation 8

2.1 ATAC-seq quality control 8
2.2 ChIP-seq quality control 9
2.3 ATAC-seq peak classification 11

3 Regulatory region conservation 13

4 Synteny 17

4.1 GENESPACE based synteny analysis 17
4.2 Synteny breakpoint inspection 18
4.3 Orthofinder based synteny analysis 19
4.4 ODP based synteny analysis 22
4.5 MALG conservation 30

5 Genome annotations 33

Part I. Transcriptome assembly pipeline 33

5.1 Nanopore read pre-processing 33
5.2 Illumina short read pre-processing 35
5.3 Preparation of RNA Poly-adenylation site data 37
5.4 Transcriptome assembly 38

Part II. High-confidence transcript filtering 42

5.5 Isoform filtering 42
5.6 Chimaeric transcript filtering 43
5.7 Fix partial 5' ORF annotations 44
5.8 Final formatting 46

6 References 48

1 Genomic resources

1.1 Assembly quality assessment

Table 1 Summary statistics for genome assemblies of the *S. mediterranea* sexual strain. dd_Smes_g4: Our previous diploid consensus assembly [1]; schMedS2: chromosome-scale scaffolding of most contigs of the dd_Smes_g4 assembly [2], schMedS3h1 and schMedS3h2: haplotype phased assemblies (this study).

	**dd_Smes_g4**	**schMedS2**	**schMedS3h1**	**schMedS3h2**
# contigs	481	4	662	432
Total length (bp)	773,939,492	763,446,644	840,173,815	819,865,861
GC (%)	29.63	29.6	29.59	29.59
N50 (bp)	3,854,845	265,042,666	270,168,396	268,961,546
chr-scaffold (%)	-	99	95	96
unplaced (Mb)	-	10.5	42	32.8

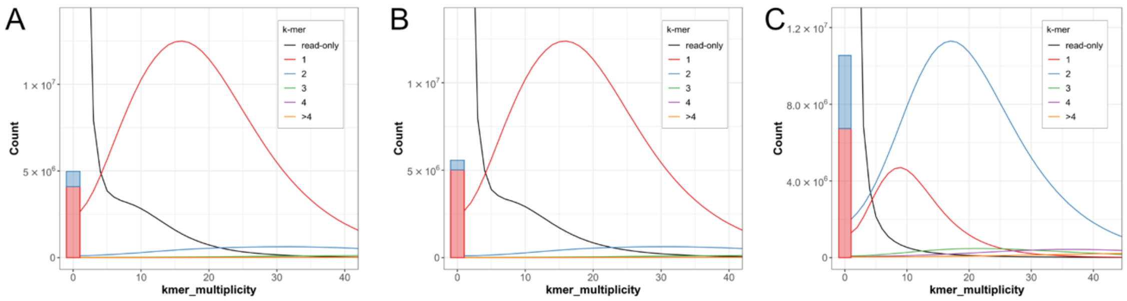

Figure 1 Representative ‘cn-spectra’ plots from a merqury k-mer analysis of the short-read dataset SRR959588. Density plots show the coverage of distinct k-mers and their copy number variation. A: schMedS3h1; B: schMedS3h2; C: schMedS3BH. Read-only k-mers with low multiplicity (peak on the left of each plot) represent errors in the short-read data. Peaks with high multiplicity represent genuine k-mers present in the assemblies. A and B show a shoulder of read-only peaks with higher than error multiplicity representing haplotype-specific k-mers absent in the respective assembly. Panel C shows that these k-mers are well represented in schMedS3BH, leading to the increase in completeness of the diploid assembly.

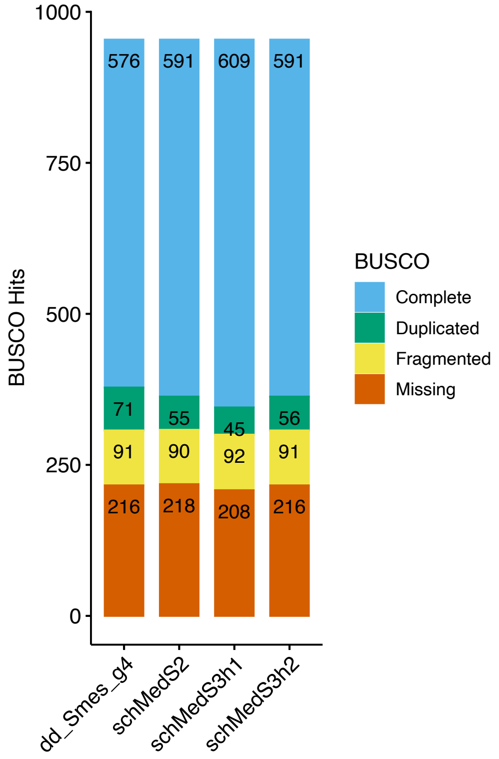

Figure 2 BUSCO scores of our previous assembly dd_Smes_g4, the schMedS2 assembly, and the two haplotypes of the new phased assembly, demonstrating increased completeness in the new assembly.

1.2 Comparison to schMedS2

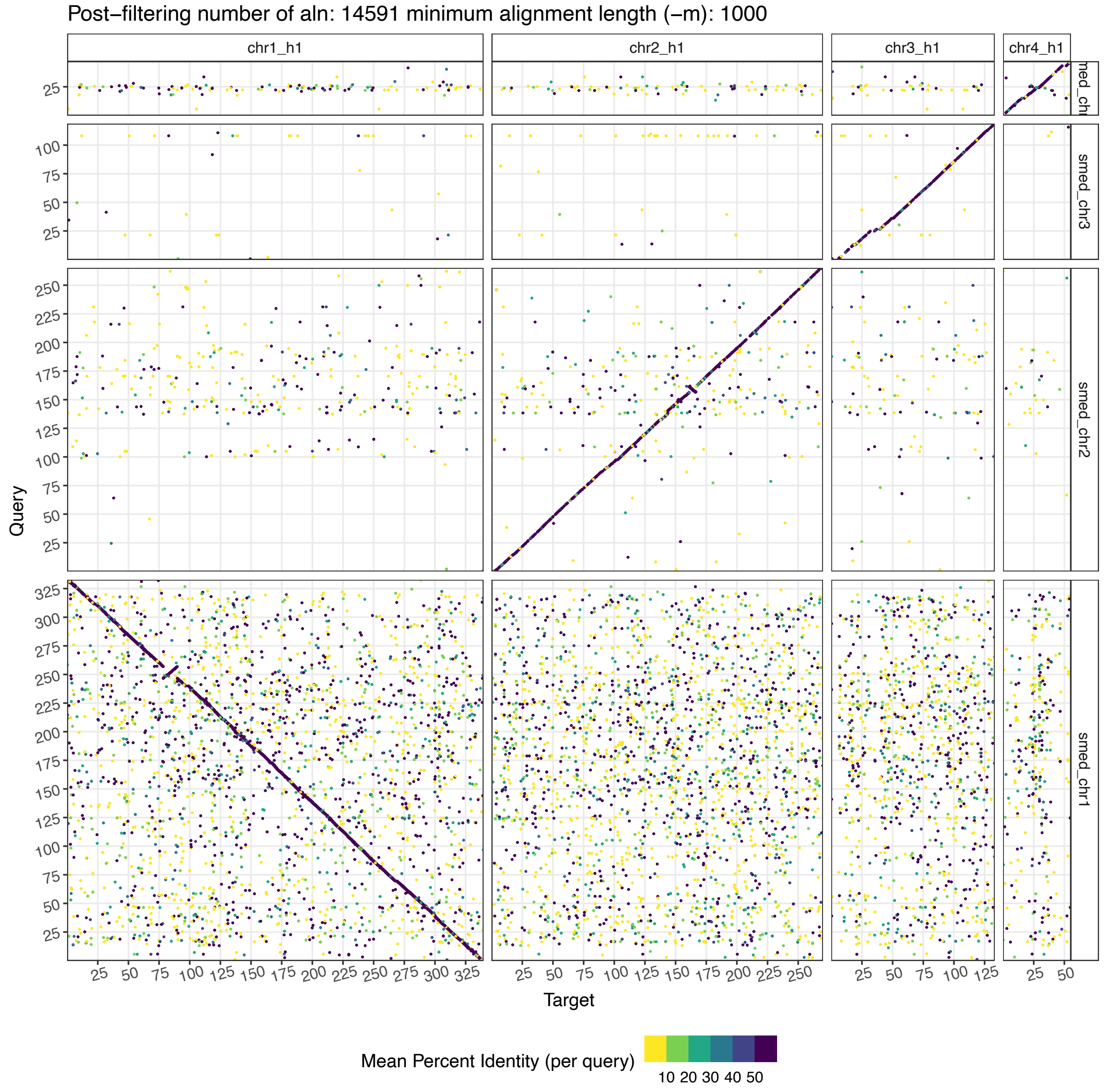

Figure 3 Dotplot alignment between schMedS3h1 from this study and schMedS2 from [2]. Dots represent alignments of at least 1000bp and the color indicates alignment quality.

1.3 Phasing efficiency

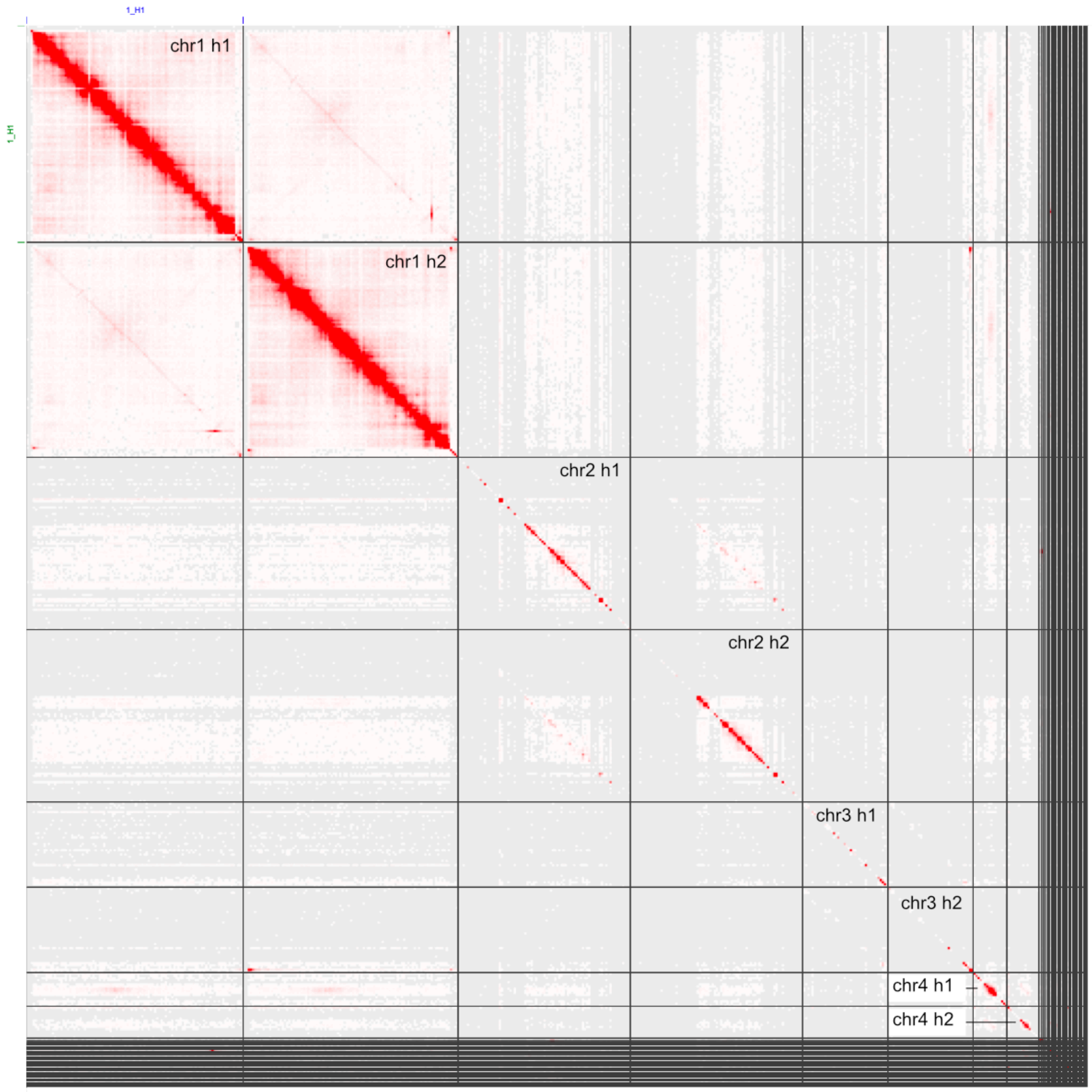

Figure 4 HiC map of the diploid assembly schMedS3BH showing a high number of uniquely mapping (red cells) on Chromosome 1, a large proportion of Chromosome 2 and Chromosome 4, and only few regions of Chromosome 3.

1.4 Gene annotation benchmarking

Table 2 Summary of the 19 gene sequences deposited in NCBI that were not detected in the S3BH annotation. Two genes could not be mapped, likely due to assembly gaps. The other 14 mapped uniquely to the assembly and eight of them were present in some of our previous annotations (SMEST, dd_Smed_v6, or dd_Smes_v1), indicating errors in the S3BH gene preditions.

Missing gene	Annotation	Map location	Predicted in x other annotations	Category
KT163545.1	Smed slc15a-9 (slc15a-9)	no hit	n.a.	Assembly gap
KX018976.1	Smed NPYR-16 (npyr-16)	no hit	n.a.	Assembly gap
BK007012.1	TPA_inf: Smed cerebral peptide prohormone like-1	unique	2	missing prediction
BK007023.1	TPA_inf: Smed secreted peptide prohormone-12	unique	1	missing prediction
FJ471488.1	Smed noggin-like protein 6 (nlg6)	unique	3	missing prediction
FJ588606.1	Smed cyclinB-like protein	unique	0	missing prediction
KT163558.1	Smed slc16a-13 (slc16a-13)	unique	0	missing prediction
KT163560.1	Smed slc16a-15 (slc16a-15)	unique	0	missing prediction
KT163565.1	Smed slc16a-20 (slc16a-20)	unique	0	missing prediction
KT163605.1	Smed slc22a-7 (slc22a-7)	unique	3	missing prediction
KT163651.1	Smed slc25a-33 (slc25a-33)	unique	0	missing prediction
KT163655.1	Smed slc26a-1 (slc26a-1)	unique	3	missing prediction
KT163750.1	Smed slc47a-3 (slc47a-3)	unique	0	missing prediction
KX018920.1	Smed GCR123 (gcr123)	unique	2	missing prediction
KX018937.1	Smed GCR141 (gcr141)	unique	1	missing prediction
KX018982.1	Smed NPYR-8 (npyr-8)	unique	2	missing prediction
AP017710.1	NADH dehydrogenase subunit 6	Mitochondrial	n.a.	mitochondrial
JX010507.1	Smed_01154_V2 cytochrome c oxidase subunit III	Mitochondrial	n.a.	mitochondrial
JX010579.1	Smed_04444_V2 hypothetical protein	Mitochondrial	n.a.	mitochondrial

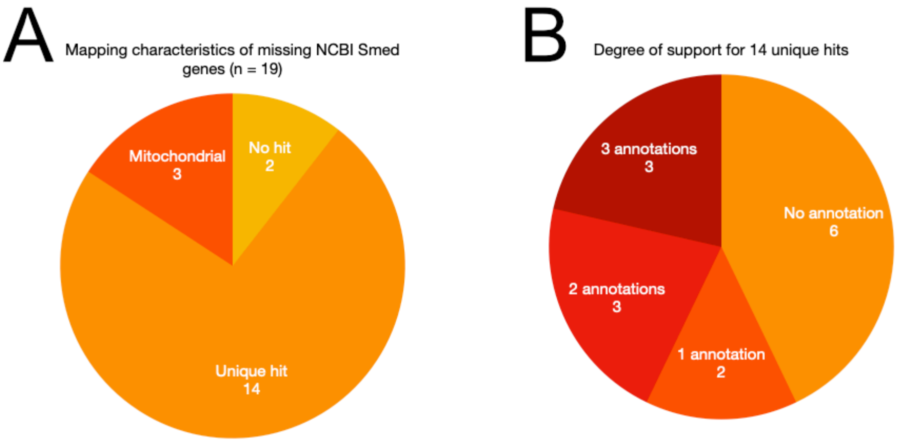

Figure 5 Details on the 19 transcript sequences deposited in NCBI that were not present in the S3BH annotation. **A** Mapping characteristic of the missing transcripts. **B** Details on whether the 14 uniquely mapped transcripts were present in some of the previous annotations (SMEST, dd_Smed_v6, or dd_Smes_v1). Due to the large fraction of non-characterized EST sequences in this data set, the transcripts with little- or no prior evidence likely represent non-coding transcripts that were not a specific focus of the current annotation effort. Note that the mitochondrial genome is not included in the schMedS3 assemblies.

1.5 Chimeric gene annotations

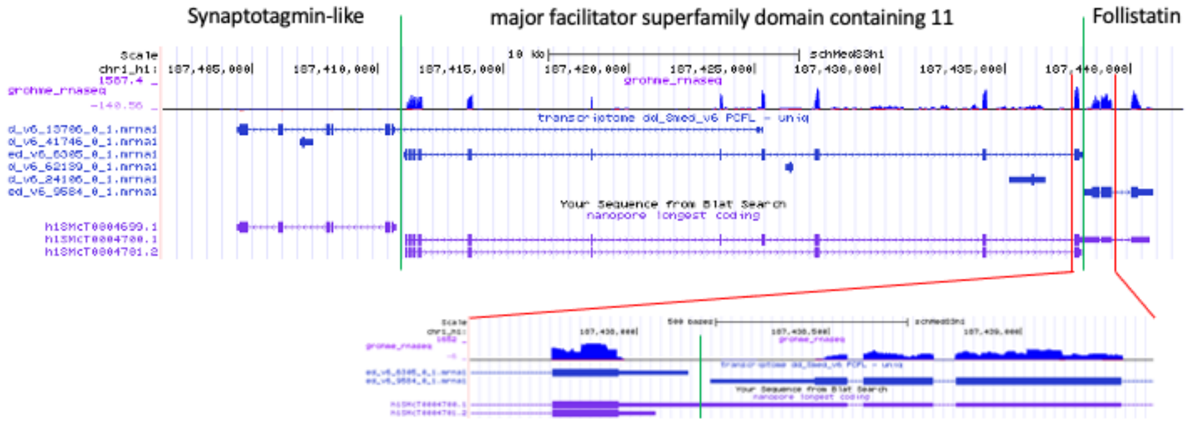

Figure 6 UCSC genome browser view showing the chimeric merger of the gene annotations of a *frz receptor* (delimited by green bounding lines) and the Activin inhibitor *follistatin* immediately downstream in the new annotations (h1SMcT*; purple, bottom tracks), but not in the previous dd_v6 transcriptome (blue, above). Note the extremely close proximity, same strandedness and similar RNA-seq coverage of both genes/transcripts, making this a particularly challenging annotation problem.

2 Regulatory element annotation

2.1 ATAC-seq quality control

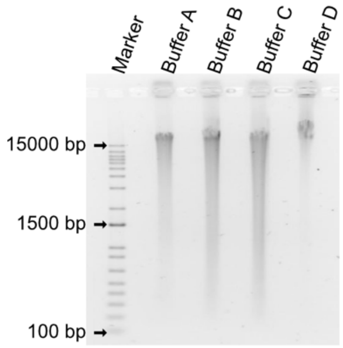

Figure 7 Impact of buffer composition on DNA integrity using native samples. Buffer A (10mM Tris- HCl pH7.5, 10mM NaCl, 3mM MgCl2, 0.1% Igepal), Buffer B (10mM HEPES- NaOH pH7.9, 1.5mM MgCl, 10 mM KCl, 1mM EDTA, 0.05% Igepal, 0.5 mM DTT (added right before use)), Buffer C: (10mM HEPES- NaOH pH7.9, 60 mM KCl, 1mM EDTA, 0.05% Igepal, 0.5 mM DTT (added right before use)) and Buffer D (10mM Tris-HCl pH7.5, 10mM NaCl, 3mM MgCl2, 0.1% Igepal CA-630, 0.5mM Spermine, 0.25mM Spermidine, 0.5 mM DTT (added right before use)). Using buffer D resulted in sufficient isolation of high molecular weight DNA.

2.2 ChIP-seq quality control

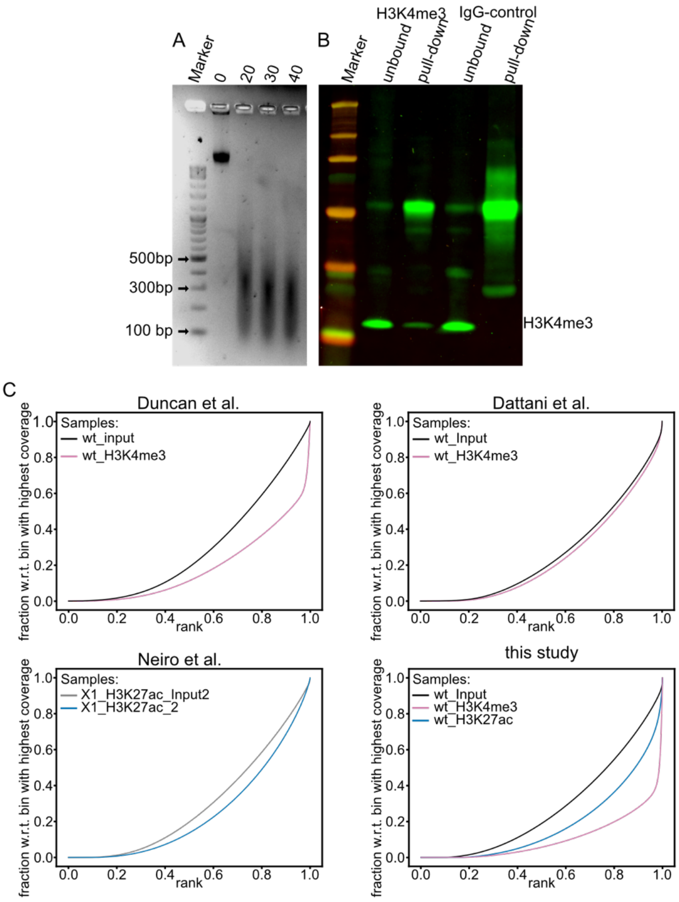

Figure 8 A Identification of optimal sonication settings for chromatin fragmentation using the Covaris S2 sonicator with AFA tubes. (5% Duty Cycle, Intensity 4, 200 Cycles/Burst, 30 sec/cycle). Displayed are DNA samples from crosslinked nuclei without sonication (0), 20 cycles, 30 cycles and 40 cycles. B: Western blot validation of H3K4me3 ChIP-seq pulldown. H3K4me3 pulldown enriched for protein of interest (left), while IgG control did not show any H3K4me3 enrichment (right), confirming the specificity of the assay. The blot was probed with the same H3K4me3 AB as used for the pull-down, which is why the IgG bands are visible (intense bands in all pull-down lanes). C Fingerprint plot displaying the degree of ChIP signal enrichment in the indicated published data sets and this study. A diagonal line indicates uniform read distribution along the genome (no enrichment/background), while a steep curve indicates strong enrichment. Accordingly, the signal/noise ratios of this study's ChIP-seq data is at least en par with the published data.

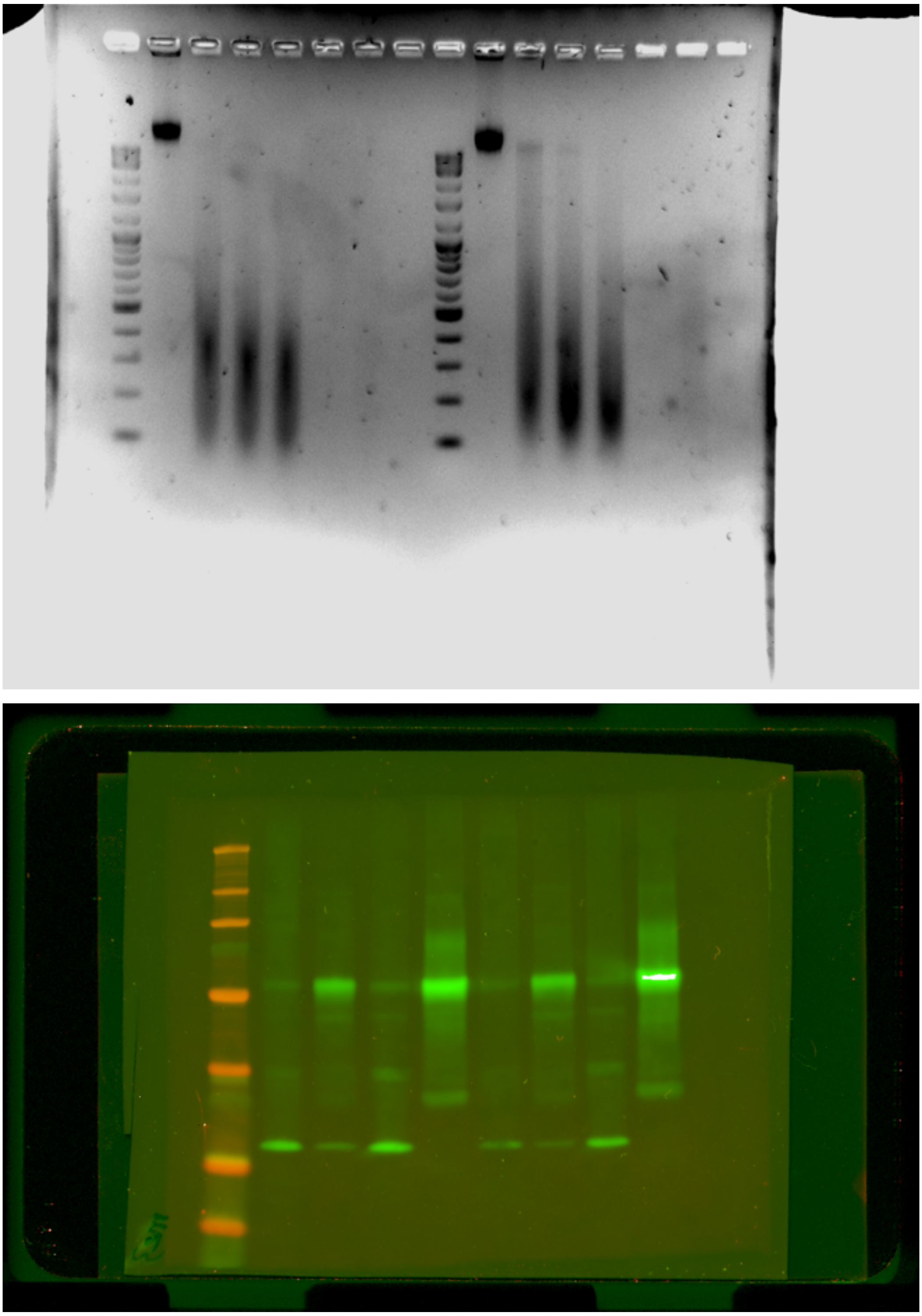

Figure 9 Uncropped and unedited gel images supporting Figure 8.

2.3 ATAC-seq peak classification

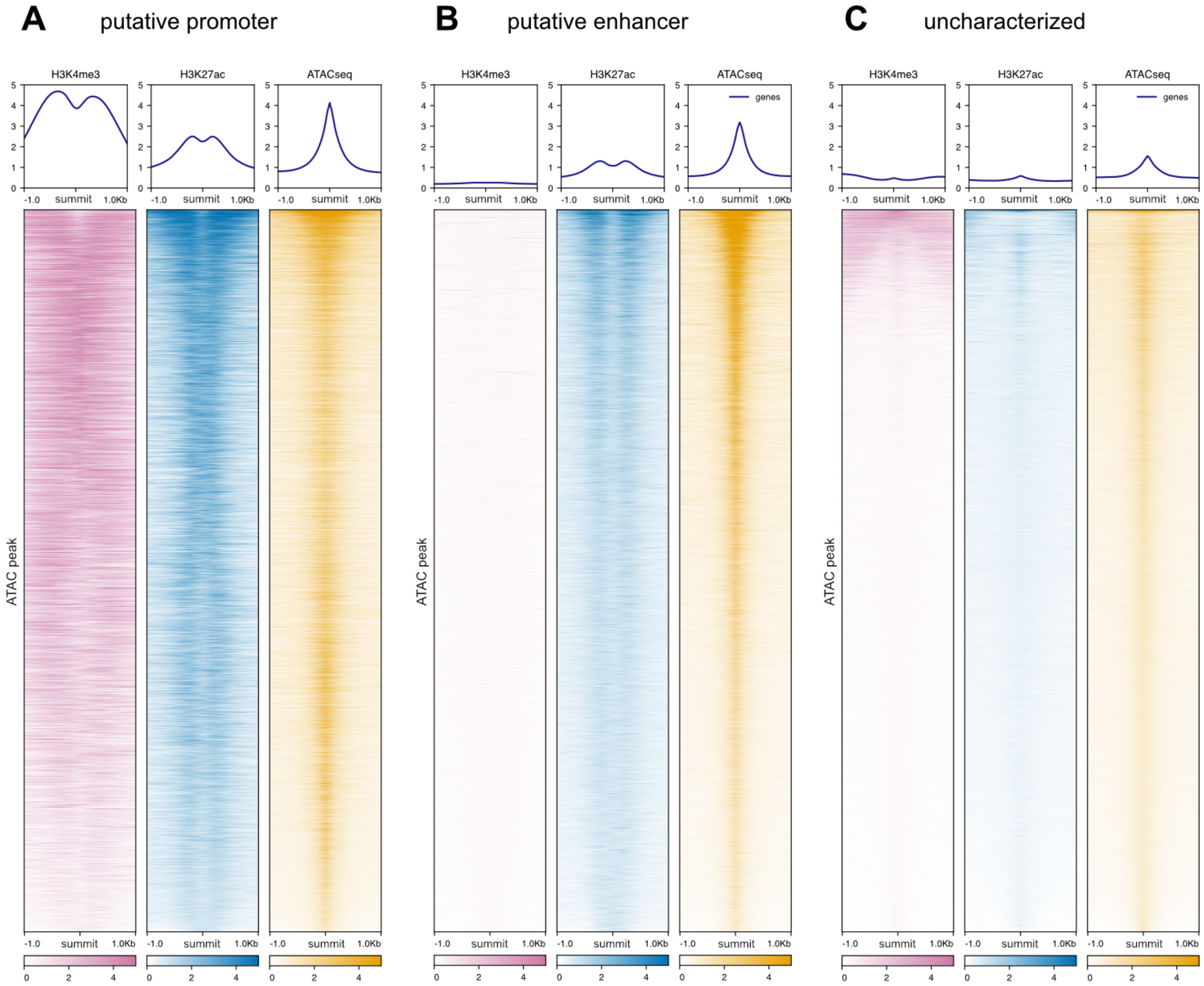

Figure 10 Heatmap of ChIP signals at the summits of ATAC-seq peaks. **A**: H3K4me3 and H3K27ac signal at the summits of putative promoters. **B**: H3K4me3 and H3K27ac signal at the summits of putative enhancers. **C**: H3K4me3 and H3K27ac signal at the summits of the remaining uncharacterized ATAC-seq peaks.

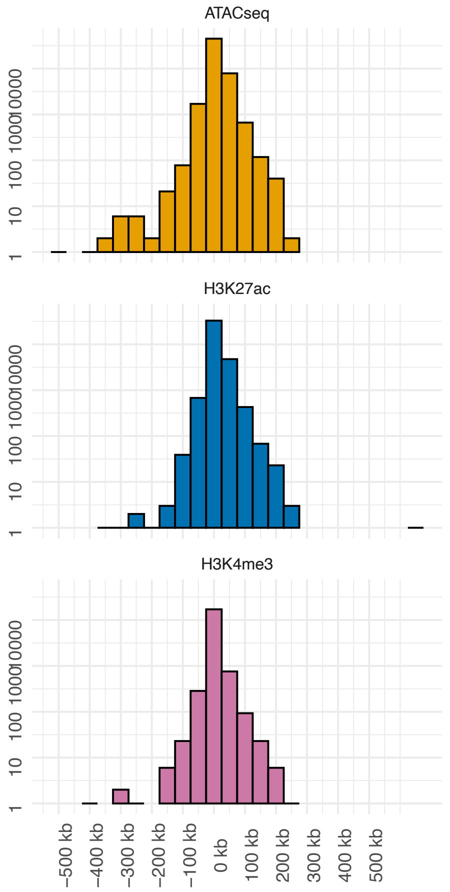

Figure 11 Histogram of the distance (from top to bottom) of ATAC-seq peaks, H3K27ac peaks, and H3K4me3 peaks to the closest TSS in *S. mediterranea*. The median distance to the TSS were 3599 bp, 1693 bp, and 0 bp for ATAC-seq peaks, H3K27ac peaks, and H3K4me3 peaks, respectively.

Table 3 Genomic location of ATAC-seq peaks depending on their CHIP-intersect classification. Annotations are based on the closest gene. Application of a Chi-square test showed that the annotations were not equally distributed across the categories, with putative promoter annotations enriched within 1 kb of the TSS, putative enhancers in introns, exons and distal intergenic regions and uncharacterized peaks in introns and distal intergenic regions. The broad match with expectations in the former two cases confirms the utility of our approach. A two-sided Z-score-based post-hoc tests with a Bonferroni correction for multiple testing to determine the enrichment of element types. Corrected p-values < 10^{-16} are indicated accordingly.

	putative promoter				putative enhancer				uncharacterized			
	N	%	Z	p	N	%	Z	p	N	%	Z	p
<1kb TSS	8444	61.4	150.8	< 1e-16	454	4.3	-41.6	< 1e-16	1263	4.1	-98.1	< 1e-16
1-2kb TSS	48	0.3	-11.7	< 1e-16	101	0.9	-4	0.0019	599	1.9	13.3	< 1e-16
2-3kb TSS	39	0.3	-9.8	< 1e-16	72	0.7	-3.8	0.0037	448	1.4	11.5	< 1e-16
5UTR	877	6.4	31.5	< 1e-16	147	1.4	-9	< 1e-16	445	1.4	-20.2	< 1e-16
Exon	2044	14.9	-9.6	< 1e-16	2402	22.6	15.1	< 1e-16	5312	17	-3.6	0.0066
Intron	1066	7.7	-62.9	< 1e-16	5240	49.2	51.7	< 1e-16	9705	31.1	13.7	< 1e-16
Downstream	38	0.3	-10	< 1e-16	99	0.9	-1.1	1.0000	432	1.4	9.6	< 1e-16
Distal intergenic	1203	8.7	-61.2	< 1e-16	2130	20.0	-23.5	< 1e-16	12977	41.6	71.8	< 1e-16

3 Regulatory region conservation

Table 4 Conservation of ATAC-seq peaks depending on their ChIP-intersect characterization. Application of a Chi-square test showed that conservation was not equally distributed across the categories (X-squared = 9745.1, df = 4, p-value < 2.2e-16). We used Z-score-based post-hoc tests with a Bonferroni correction for multiple testing to determine the enrichment of element types. All comparisons were highly statistically significant (all p-values < 10^{-16}), indicating enrichment for conserved peaks in putative promoters and putative enhancers (positive Z-scores) but enrichment for no conservation in uncharacterized peaks.

	putative promoter		putative enhancer		uncharacterized	
	N	Z-score	N	Z-score	N	Z-score
highly conserved	4261	68.4	2343	28.1	963	-81.8
partially conserved	6659	22.7	6006	38	9661	-49.9
not conserved	2839	-69.4	2296	-56.7	20557	105.3
% highly conserved	31		22		3.1	
% min. partially conserved	79.4		78.4		34.1	

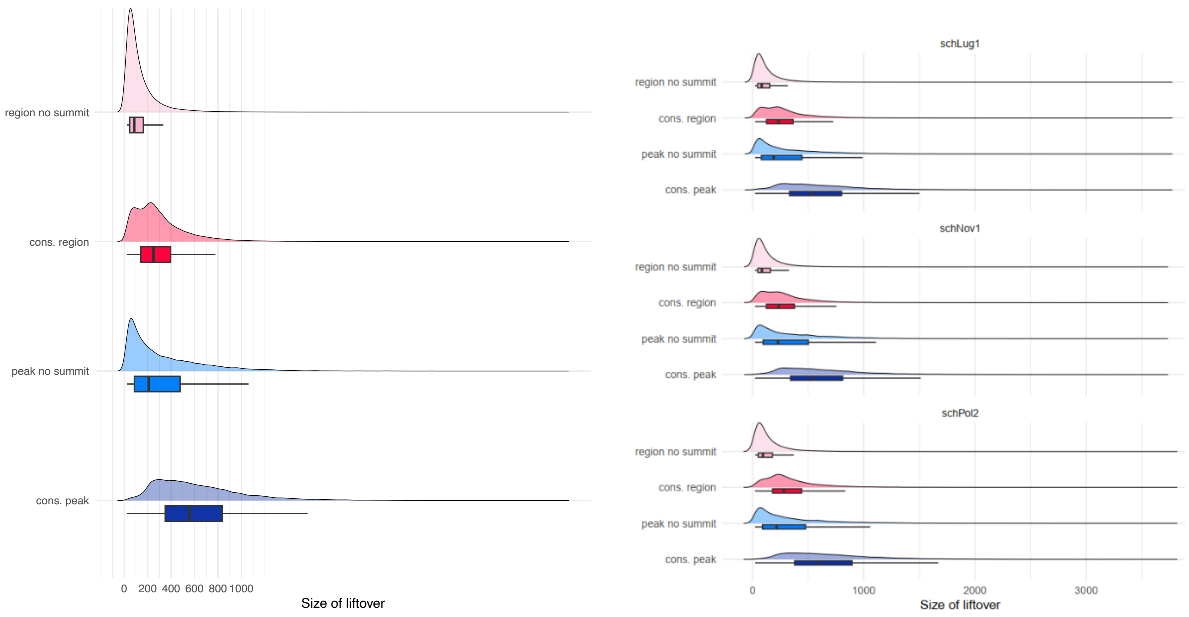

Figure 12 Size distribution of *S. mediterranea* ATAC-seq peak liftover depending on the conservation classification. Left side: Cumulative analysis of all three liftover species. Liftovers categorized as “not conserved” (region no summit and peak no summit; top), were shorter than conserved peaks (bottom). Right side: Same plot but split by species.

Table 5 Size distribution of the cumulative schMedS3h1 liftover by category from Supplemental Figure 12-left.

	median size	SD
region no summit	85	141.4
cons. region	249	246.3
peak no summit	209	329.0
cons. peak	555	410.2

Table 6 Size distributions of schMedS3h1 liftover by category and species from Supplemental Figure 12-right.

species	type	median size	SD
S. lugubris	region no summit	82	133.3
	cons. region	232	228.2
	peak no summit	190	315.8
	cons. peak	531	394.0
S. nova	region no summit	84	136.3
	cons. region	235	234.4
	peak no summit	230	328.6
	cons. peak	539	393.4
S. polychroa	region no summit	93	158.7
	cons. region	280	268.2
	peak no summit	216	346.5
	cons. peak	597	436.8

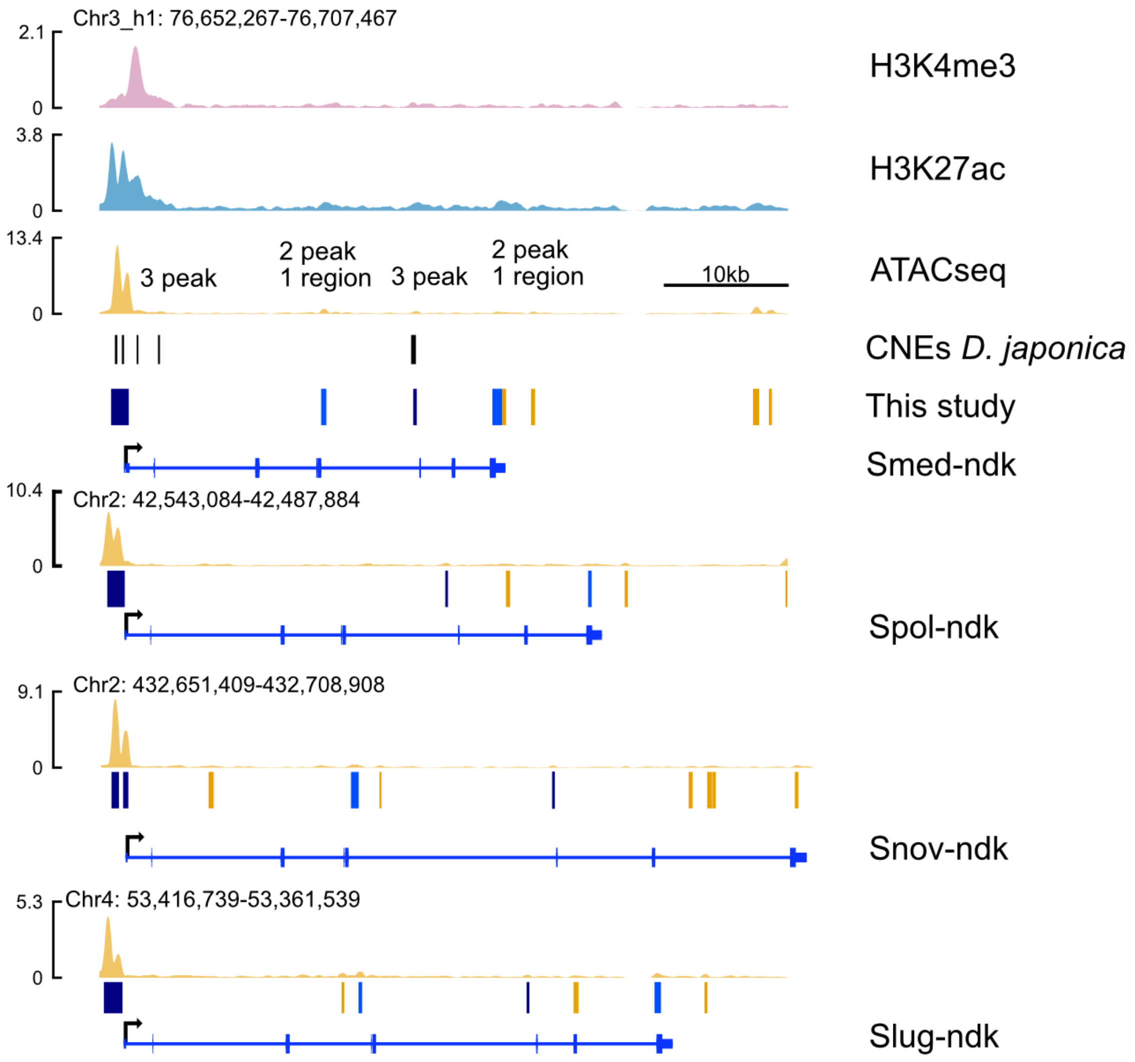

Figure 13 Example of a highly conserved regulatory elements at the *nou-darake* (*ndk*) gene locus, showing (top to bottom) the H3K4me3 ChIP, H3K27ac ChIP, and ATAC-seq signal tracks for *S. mediterranea*, the of mapping of conserved non-coding elements (CNE) previously inferred on basis of the draft genome of *Dugesia japonica* [3] , the CNEs predicted by this study, the exon/intron gene annotation of S. mediterranea ndk, followed by the ATAC-seq signal, CNE predictions and ndk gene annotations in the genomes of *S. polychroa* (schPol2), *S. nova* (schNov1), and *S. lugubris* (schLug1), all aligned by the ndk TSS. Dark blue, blue or yellow vertical bars indicate highly conserved, conserved and non-conserved ATAC-seq peaks.

4 Synteny

4.1 GENESPACE based synteny analysis

Table 7 Syntenic blocks identified between schMedS3h1 and the other assemblies using GENESPACE. Given is the number of syntenic blocks with their median, standard deviation, minimum size, maximum size, and the genome coverage in Mb and as a percentage.

genome	blocks	median (Mb)	SD (Mb)	min (Mb)	max (Mb)	block coverage (Mb)	block coverage (%)
schMedS3h2	18	12.5	59.1	0.7	192.9	797.8	97.2
schPol2	198	2.2	4.3	0.1	33.4	752.4	96.3
schNov1	166	2.1	5.8	0.2	33.4	747.5	59.7
schLug1	272	1.5	3.2	0.1	26.7	730.8	48.7

4.2 Synteny breakpoint inspection

Table 8 Enrichment analysis of 10 kb windows flanking synteny breakpoints inferred using GENESPACE. Tests are two-sided and based on comparisons to 1000 iterations of random placement of an equal number of 10 kb windows in the reference. Given are results for all transposable elements followed by the LTR/Gypsy and LINE/R2 elements since they returned the most relevant results. For a full table including all tested elements, see Additional File 2: Table S12. P values are adjusted for multiple testing using the Benjamini-Hochberg procedure and printed in bold when <0.05.

reference	target	observed	expected	SD	padj_larger
schMedS3h1	schLug1	8387	7065	204	**0.000**
schMedS3h1	schMedS3h2	921	621	56	**0.007**
schMedS3h1	schNov1	5035	4414	157	**0.008**
schMedS3h1	schPol2	6288	5283	182	**0.000**
schMedS3h2	schMedS3h1	960	668	63	**0.011**
schPol2	schMedS3h1	5913	5435	152	**0.000**
schNov1	schMedS3h1	6007	6319	474	0.998
schLug1	schMedS3h1	9884	9503	256	0.303
LTR/Gypsy					
schMedS3h1	schLug1	1741	1277	58	**0.000**
schMedS3h1	schMedS3h2	164	110	16	**0.020**
schMedS3h1	schNov1	1059	792	43	**0.000**
schMedS3h1	schPol2	1269	958	51	**0.000**
schMedS3h2	schMedS3h1	148	99	16	**0.033**
schPol2	schMedS3h1	1533	1320	64	**0.000**
schNov1	schMedS3h1	1980	1799	82	0.076
schLug1	schMedS3h1	2600	2275	76	**0.000**
LINE/R2					
schMedS3h1	schLug1	1249	664	52	**0.000**
schMedS3h1	schMedS3h2	103	59	14	**0.007**
schMedS3h1	schNov1	777	412	40	**0.000**
schMedS3h1	schPol2	1024	506	45	**0.000**
schMedS3h2	schMedS3h1	90	62	16	0.180
schPol2	schMedS3h1	22	20	11	0.708
schNov1	schMedS3h1	52	25	6	0.057
schLug1	schMedS3h1	9	11	5	0.995

4.3 Orthofinder based synteny analysis

As described in the main text, we used single-copy orthologs derived using Orthofinder to test for synteny conservation using dotplots and Chi-square tests. The results for the comparison of *S. mediterranea* haplotype 1 against the other *Schmidtea* and the parasites as well as *Schistosoma mansoni* against the same are shown below (Table 9-10). Additionally, we compared *Schmidtea* and the parasites to Amphioxus (*Branchiostoma lancelatum*), a representative of ancestral vertebrate linkage groups. Although this analysis recapitulated the previously described conservation of synteny between the cnidarian *Nematostella vectensis* and Amphioxus [4], synteny conservation was lost in comparison with members of the genus *Schmidtea* (Table 11). Interestingly, macrosynteny between Amphioxus and *S. mansoni* and the other parasites was degraded with all effect sizes <= 0.22 (Table 11), but some synteny appeared conserved for specific chromosomes (e.g., Amphioxus chromosomes 3 and 4).

Table 9 Test for macrosynteny using a chi-square test of the distribution of one-to-one orthologs between the target and query genome assemblies. Given is the number of orthologs, the degrees of freedom, the chi-squared statistic, the effect size Cramer's V which is 0 for a random distribution and 1 for a perfect correlation and the p-value calculated assuming the chi-square distribution, or 100,000 permutations of the data. P-values < 10^{-16} for the chi-square test are noted as such. For the permutation test, p-values below its precision of 10^{-5} are indicated accordingly.

target	query	orthologs	df	chisq	V	p-val	perm. p-val
schMedS3h1	schMedS3h2	11718	9	34436.9	0.99	**<1e-16**	**< 1e-05**
schMedS3h1	schLug1	9479	9	10233.4	0.6	**<1e-16**	**< 1e-05**
schMedS3h1	schPol2	9458	9	18508.7	0.81	**<1e-16**	**< 1e-05**
schMedS3h1	schNov1	9321	6	7150.1	0.62	**<1e-16**	**< 1e-05**
schMedS3h1	cloSin	3440	18	43.1	0.06	**0.000774**	**0.00087**
schMedS3h1	schMan	2602	27	45.3	0.08	0.0151	0.015
schMedS3h1	hymMic	2996	15	15.2	0.04	0.438	0.44052
schMedS3h1	taeMul	2823	15	39.3	0.07	**0.000569**	**0.00055**

Table 10 Test for macrosynteny using a chi-square test of the distribution of one-to-one orthologs between the target and query genome assemblies. Given is the number of orthologs, the degrees of freedom, the chi-squared statistic, the effect size Cramer's V which is 0 for a random distribution and 1 for a perfect correlation and the p-value calculated assuming the chi-square distribution, or 100,000 permutations of the data. P-values < 10^{-16} for the chi-square test are noted as such. For the permutation test, p-values below its precision of 10^{-5} are indicated accordingly.

target	query	orthologs	df	chisq	V	p-val	perm. p-val
schMan	schMedS3h2	2587	27	46.9	0.08	0.0102	0.01036
schMan	schPol2	2555	27	43.1	0.07	0.0255	0.02538
schMan	schNov1	2506	18	24.4	0.07	0.142	0.13971
schMan	schLug1	2572	27	54.8	0.08	0.0012	0.00129
schMan	cloSin	3075	54	10406.1	0.75	<1e-16	< 1e-05
schMan	hymMic	2547	45	7789.5	0.78	<1e-16	< 1e-05
schMan	taeMul	2365	45	6998.4	0.77	<1e-16	< 1e-05

Table 11 Test for macrosynteny using a chi-square test of the distribution of one-to-one orthologs between the target and query genome assemblies. Given is the number of orthologs, the degrees of freedom, the chi-squared statistic, the effect size Cramer's V which is 0 for a random distribution and 1 for a perfect correlation and the p-value calculated assuming the chi-square distribution, or 100,000 permutations of the data. P-values < 10^{-16} for the chi-square test are noted as such. For the permutation test, p-values below its precision of 10^{-5} are indicated accordingly.

target	query	orthologs	df	chisq	V	p-val	perm. p-val
BraLan	NemVec	5624	252	32122.4	0.64	**<1e-16**	< 1e-05
BraLan	schMedS3h1	3918	54	75.5	0.08	**0.0282**	0.0282
BraLan	schMedS3h2	3890	54	77	0.08	**0.0217**	0.0210
BraLan	schPol2	3788	54	67.2	0.08	0.107	0.1061
BraLan	schNov1	3760	36	41.6	0.07	0.241	0.2403
BraLan	schLug1	3831	54	67.7	0.08	0.0994	0.0993
BraLan	cloSin	3289	108	988.1	0.22	<1e-16	< 1e-05
BraLan	schMan	2577	162	1060.4	0.21	<1e-16	< 1e-05
BraLan	hymMic	2953	90	575	0.2	<1e-16	< 1e-05
BraLan	taeMul	2883	90	626.6	0.21	<1e-16	< 1e-05

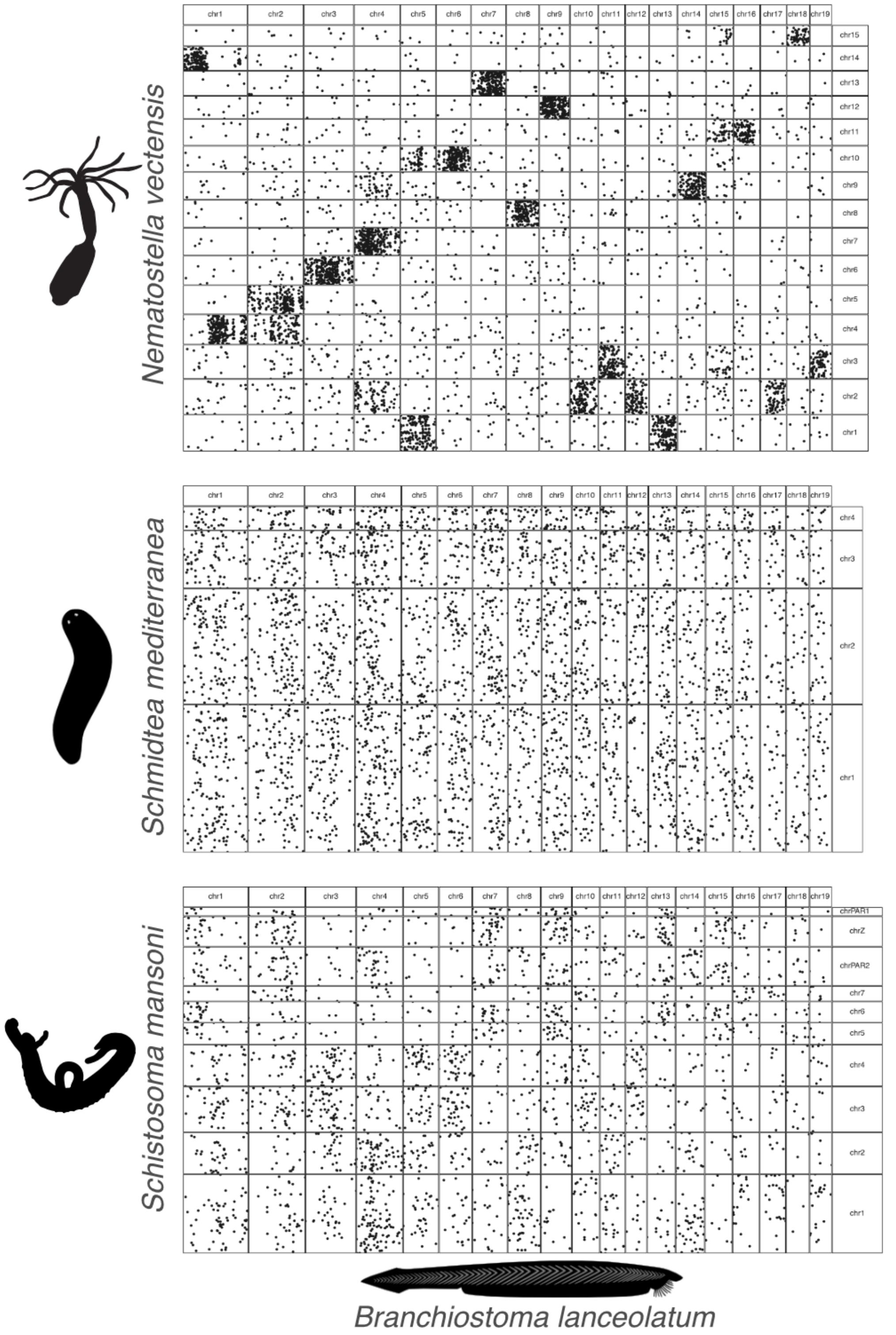

Figure 14 Oxford dotplot showing orthologous genes between Amphioxus as representative of vertebrate ancestral linkage groups and the cnidarian *Nematostella vectensis*, *Schmidtea mediterranea* haplotype 1, and the parasitic flatworm *Schistosoma mansoni*.

4.4 ODP based synteny analysis

To evaluate the synteny among the investigated genomes, we utilized the ODP tool. In the comparisons among the parasites, there was a marked conservation of synteny, as evidenced by significant p-values from Fisher's exact tests (Table 12). This conservation was evident in the pronounced clustering of orthologs by chromosomes, and even within chromosomal segments. Similarly, within *Schmidtea*, we observed substantial synteny conservation, with by significant p-values from Fisher's exact tests (Table 13) and the distinct clustering of orthologs by chromosome and gene order.

However, in pairwise comparisons between *Schmidtea* and the parasites, there was no evident clustering of orthologs by chromosomes (Table 14). The results of Fisher's exact tests indicated no association for cloSin. For taeMul, an association was observed between LG6 and chromosome 2 of both schNov1 and schLug1, though this was based on a limited gene set and was accompanied by a high p-value (Table 14). For hymMic, a more pronounced association was found: scaffold HMN_01_pilon aligned with a chromosome in schMedS3h1, schNov1, and schPol2, while HMN_06_pilon was associated with chr4 in schMedS3h1. Despite these associations involving a greater number of genes, the p-values remained elevated, consistent with the observed lack of clear clustering in the dotplot. For schMan a similar picture emerges, but with statistically significant associations of at least one chromosome with each *Schmidtea* species (Table 14). Lastly, there was no significant association of any Machtxv2 scaffold with a chromosome of any other species included in this study.

Table 12 Results of one-sided test for the hypergeometric distribution via Fisher's exact tests for synteny conservation between the parasite genomes in the study. P-values have been adjusted for multiple testing using the Bonferroni method.

genome1	genome2	scaf1	scaf2	p-value	count
cloSin	hymMic	1	HMN_01_pilon	7.66E-243	1177
cloSin	hymMic	1	HMN_03_pilon	9.36E-216	664
cloSin	hymMic	2	HMN_04_pilon	2.42E-133	610
cloSin	hymMic	2	HMN_02_pilon	1.53E-76	475
cloSin	hymMic	2	HMN_05_pilon	9.67E-72	447
cloSin	hymMic	1	HMN_06_pilon	3.46E-11	303
cloSin	hymMic	3	HMN_04_pilon	1.53E-159	238
cloSin	hymMic	5	HMN_02_pilon	5.00E-169	232
cloSin	hymMic	4	HMN_01_pilon	3.53E-90	228
cloSin	hymMic	6	HMN_05_pilon	6.59E-158	212
cloSin	hymMic	7	HMN_06_pilon	1.11E-148	170
cloSin	schMan	1	SM_V9_1	1.15E-29	905
cloSin	schMan	2	SM_V9_3	0.00E+00	820
cloSin	schMan	1	SM_V9_2	1.92E-294	817
cloSin	schMan	1	SM_V9_4	7.26E-265	787

cloSin	schMan	2	SM_V9_ZSR	0.00E+00	658
cloSin	schMan	2	SM_V9_PAR2	1.56E-46	433
cloSin	schMan	1	SM_V9_6	5.22E-123	410
cloSin	schMan	3	SM_V9_1	9.05E-210	355
cloSin	schMan	5	SM_V9_PAR2	2.69E-288	311
cloSin	schMan	6	SM_V9_1	1.13E-180	305
cloSin	schMan	4	SM_V9_5	0.00E+00	305
cloSin	schMan	7	SM_V9_7	0.00E+00	260
cloSin	schMan	2	SM_V9_PAR1	4.29E-75	163
cloSin	taeMul	1	LG1	3.80E-78	1638
cloSin	taeMul	2	LG3	7.86E-178	406
cloSin	taeMul	3	LG1	9.76E-21	220
cloSin	taeMul	5	LG1	2.75E-21	207
cloSin	taeMul	4	LG5	1.56E-280	207
cloSin	taeMul	6	LG4	9.03E-255	186
cloSin	taeMul	1	LG7	6.68E-35	151
cloSin	taeMul	7	LG2	8.63E-215	144
cloSin	taeMul	1	LG6	5.15E-31	120
hymMic	schMan	HMN_03_pilon	SM_V9_1	0.00E+00	675
hymMic	schMan	HMN_04_pilon	SM_V9_3	0.00E+00	626
hymMic	schMan	HMN_01_pilon	SM_V9_4	6.29e-313	602
hymMic	schMan	HMN_01_pilon	SM_V9_2	1.03E-305	593
hymMic	schMan	HMN_02_pilon	SM_V9_PAR2	0.00E+00	523
hymMic	schMan	HMN_05_pilon	SM_V9_ZSR	5.85E-159	314
hymMic	schMan	HMN_06_pilon	SM_V9_6	1.90E-292	288
hymMic	schMan	HMN_04_pilon	SM_V9_1	5.22E-03	274
hymMic	schMan	HMN_01_pilon	SM_V9_5	2.31E-89	228
hymMic	schMan	HMN_05_pilon	SM_V9_1	3.97E-05	227
hymMic	schMan	HMN_02_pilon	SM_V9_ZSR	4.26E-29	172
hymMic	schMan	HMN_06_pilon	SM_V9_7	2.10E-137	159
hymMic	schMan	HMN_05_pilon	SM_V9_PAR1	3.15E-65	104
hymMic	taeMul	HMN_01_pilon	LG1	8.86E-24	1314
hymMic	taeMul	HMN_04_pilon	LG1	2.96E-146	1001
hymMic	taeMul	HMN_02_pilon	LG1	5.37E-105	828
hymMic	taeMul	HMN_03_pilon	LG1	2.13E-104	776
hymMic	taeMul	HMN_05_pilon	LG3	0.00E+00	527
hymMic	taeMul	HMN_05_pilon	LG4	7.78E-227	283
hymMic	taeMul	HMN_01_pilon	LG5	7.48E-143	282
hymMic	taeMul	HMN_06_pilon	LG2	5.17E-259	213
hymMic	taeMul	HMN_06_pilon	LG6	1.35E-197	168

schMan	taeMul	SM_V9_1	LG1	2.53E-05	850
schMan	taeMul	SM_V9_3	LG1	9.61E-69	542
schMan	taeMul	SM_V9_2	LG1	2.59E-62	533
schMan	taeMul	SM_V9_4	LG1	3.69E-62	532
schMan	taeMul	SM_V9_PAR2	LG1	3.04E-28	475
schMan	taeMul	SM_V9_ZSR	LG3	3.09E-204	282
schMan	taeMul	SM_V9_5	LG5	6.99E-276	200
schMan	taeMul	SM_V9_1	LG4	1.14E-91	200
schMan	taeMul	SM_V9_7	LG2	4.91E-223	149
schMan	taeMul	SM_V9_6	LG6	3.96E-186	119
schMan	taeMul	SM_V9_PAR1	LG3	6.47E-74	92

Table 13 Results of one-sided test for the hypergeometric distribution via Fisher's exact tests for synteny conservation between the *Schmidtea* genomes in the study. schMedS3h2 was omitted for readability since it had qualitatively identical results to schMedS3h1. P-values have been adjusted for multiple testing using the Bonferroni method.

genome1	genome2	scaf1	scaf2	p-value	count
schLug1	schMedS3h1	chr2	chr1_h1	0.00E+00	3104
schLug1	schMedS3h1	chr1	chr2_h1	1.56E-55	2508
schLug1	schMedS3h1	chr3	chr2_h1	0.00E+00	2051
schLug1	schMedS3h1	chr2	chr4_h1	0.00E+00	1413
schLug1	schMedS3h1	chr1	chr3_h1	2.66E-33	1403
schLug1	schMedS3h1	chr4	chr3_h1	0.00E+00	1069
schLug1	schNov1	chr2	chr2	0.00E+00	4621
schLug1	schNov1	chr1	chr1	0.00E+00	4410
schLug1	schNov1	chr3	chr1	0.00E+00	2076
schLug1	schNov1	chr1	chr3	0.00E+00	1681
schLug1	schNov1	chr4	chr2	0.00E+00	1033
schLug1	schPol2	chr2	chr3	0.00E+00	3096
schLug1	schPol2	chr1	chr1	5.44E-64	2563
schLug1	schPol2	chr1	chr4	0.00E+00	2320
schLug1	schPol2	chr3	chr1	0.00E+00	2055
schLug1	schPol2	chr2	chr2	3.67E-12	1476
schLug1	schPol2	chr4	chr2	0.00E+00	1056
schMedS3h1	schNov1	chr2_h1	chr1	0.00E+00	4495
schMedS3h1	schNov1	chr1_h1	chr2	2.05E-219	3168
schMedS3h1	schNov1	chr1_h1	chr3	0.00E+00	1664
schMedS3h1	schNov1	chr4_h1	chr2	0.00E+00	1404
schMedS3h1	schNov1	chr3_h1	chr1	3.67E-16	1311
schMedS3h1	schNov1	chr3_h1	chr2	8.32E-05	1084
schMedS3h1	schPol2	chr2_h1	chr1	0.00E+00	4619
schMedS3h1	schPol2	chr1_h1	chr3	0.00E+00	3172
schMedS3h1	schPol2	chr3_h1	chr2	0.00E+00	2515
schMedS3h1	schPol2	chr1_h1	chr4	0.00E+00	2342
schMedS3h1	schPol2	chr4_h1	chr2	0.00E+00	1439
schMedS3h1	schMedS3h2	chr1_h1	chr1_h2	0.00E+00	6670
schMedS3h1	schMedS3h2	chr2_h1	chr2_h2	0.00E+00	6171
schMedS3h1	schMedS3h2	chr3_h1	chr3_h2	0.00E+00	3458
schMedS3h1	schMedS3h2	chr4_h1	chr4_h2	0.00E+00	1646
schNov1	schPol2	chr1	chr1	0.00E+00	4537
schNov1	schPol2	chr2	chr3	0.00E+00	3160
schNov1	schPol2	chr2	chr2	1.67E-271	2488
schNov1	schPol2	chr3	chr4	0.00E+00	1634

Table 14 Results of one-sided test for the hypergeometric distribution via Fisher's exact tests for synteny conservation between the *Schmidtea* genomes and the parasite genomes in the study. schMedS3h2 was omitted for readability since it had qualitatively identical results to schMedS3h1. P-values have been adjusted for multiple testing using the Bonferroni method.

genome1	genome2	scaf1	scaf2	p	count
cloSin	schLug1	NA	NA	NA	NA
cloSin	schMedS3h1	NA	NA	NA	NA
cloSin	schNov1	NA	NA	NA	NA
cloSin	schPol2	NA	NA	NA	NA
hymMic	schLug1	NA	NA	NA	NA
hymMic	schMedS3h1	HMN_01_pilon	chr3_h1	6.04E-03	259
hymMic	schMedS3h1	HMN_06_pilon	chr4_h1	6.03E-03	64
hymMic	schNov1	HMN_01_pilon	chr3	1.30E-02	171
hymMic	schPol2	HMN_01_pilon	chr4	1.09E-02	238
schMan	schLug1	SM_V9_5	chr1	1.70E-02	130
schMan	schLug1	SM_V9_1	chr2	2.09E-02	425
schMan	schLug1	SM_V9_6	chr2	3.15E-05	129
schMan	schLug1	SM_V9_4	chr3	1.48E-02	118
schMan	schMedS3h1	SM_V9_6	chr4_h1	1.17E-05	57
schMan	schNov1	SM_V9_6	chr2	4.63E-04	148
schMan	schNov1	SM_V9_4	chr3	2.89E-02	93
schMan	schPol2	SM_V9_1	chr3	4.01E-02	297
schMan	schPol2	SM_V9_5	chr4	1.26E-04	66
taeMul	schLug1	LG6	chr2	1.63E-02	47
taeMul	schMedS3h1	NA	NA	NA	NA
taeMul	schNov1	LG6	chr2	1.82E-02	55
taeMul	schPol2	NA	NA	NA	NA

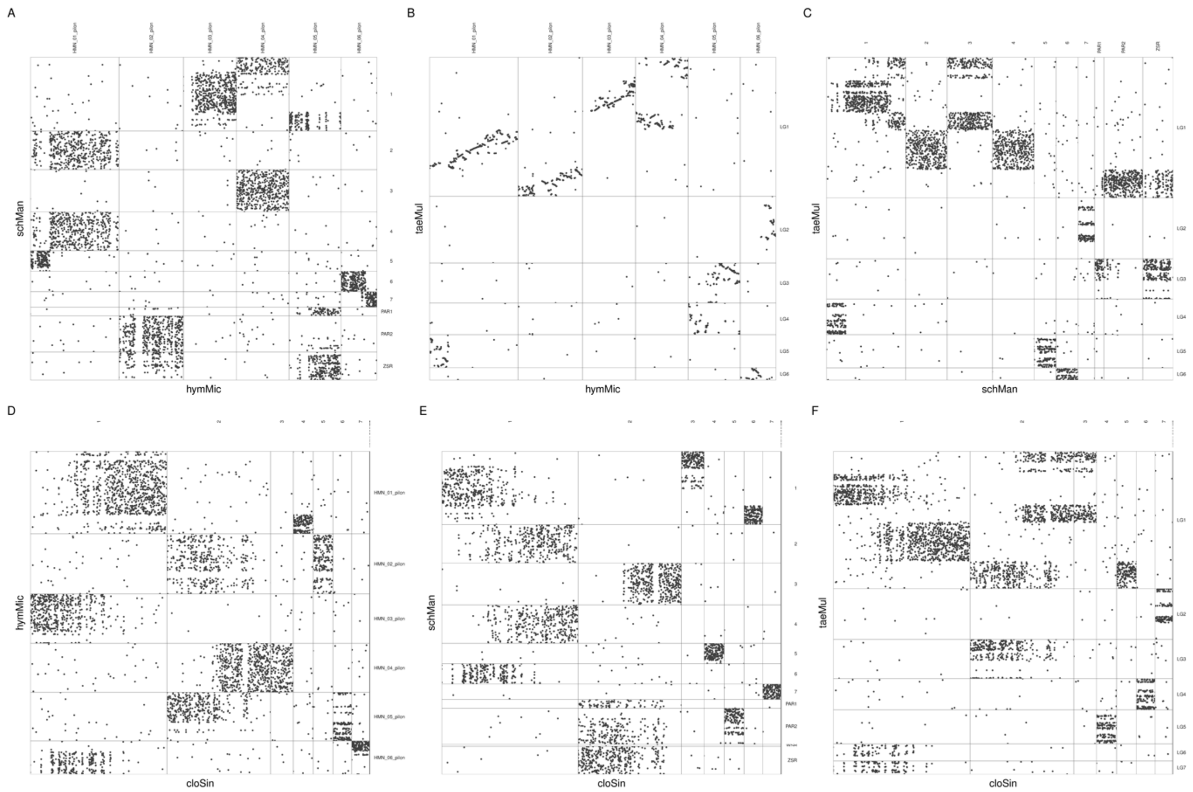

Figure 15 Oxford dotplot between the parasites species included in this study. A *Hymenolepis microstoma* vs *Schistosoma mansoni*. B *Hymenolepis microstoma* vs *Taenia multiceps*., C *Schistosoma mansoni* vs *Taenia multiceps*. D *Clonorchis sinensis* vs *Hymenolepis microstoma*. E *Clonorchis sinensis* vs *Schistosoma mansoni*. F *Clonorchis sinensis* vs *Taenia multiceps*.

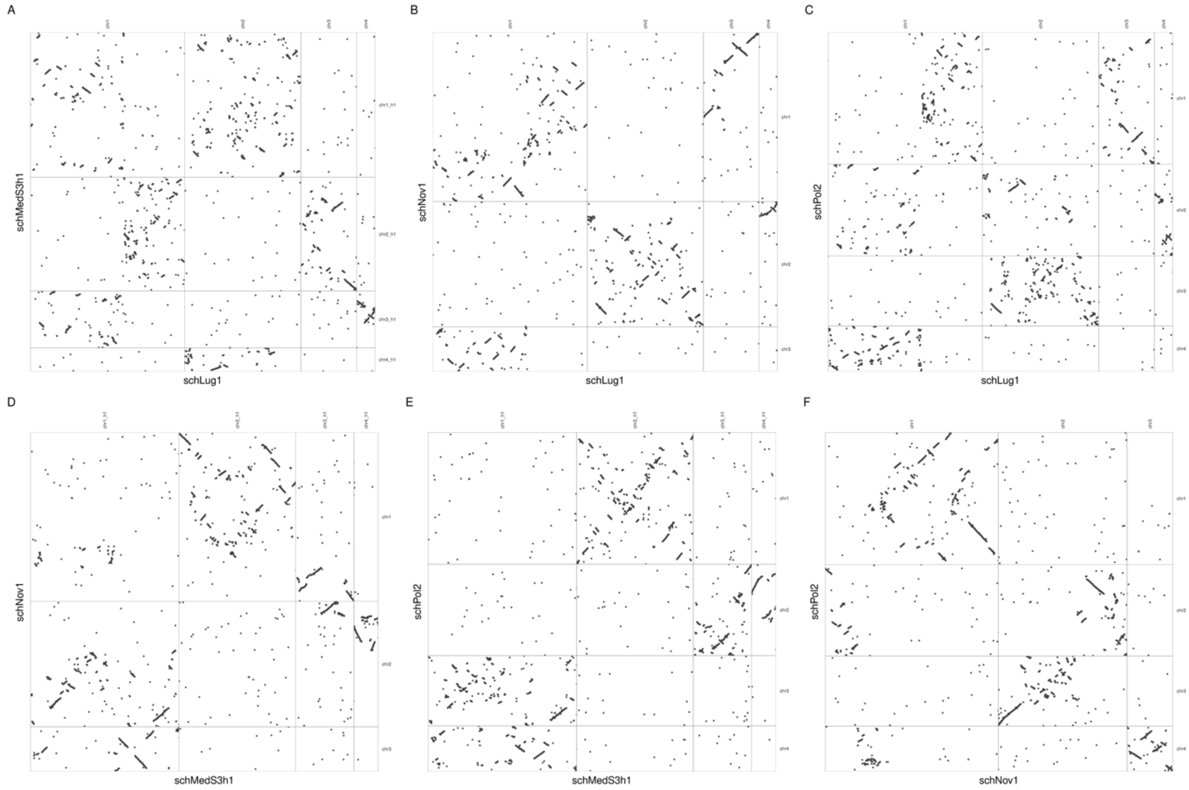

Figure 16 Oxford dotplots between the *Schmidtea* species included in this study. A *S. lugubris* vs *S. mediterranea* haplotype 1. B *S. lugubris* vs *S. nova*. C *S. lugubris* vs *S. polychroa*. D *S. mediterranea* vs *S. nova*. E, *S. mediterranea* vs *S. polychroa*. F *S. nova* vs *S. polychroa*.

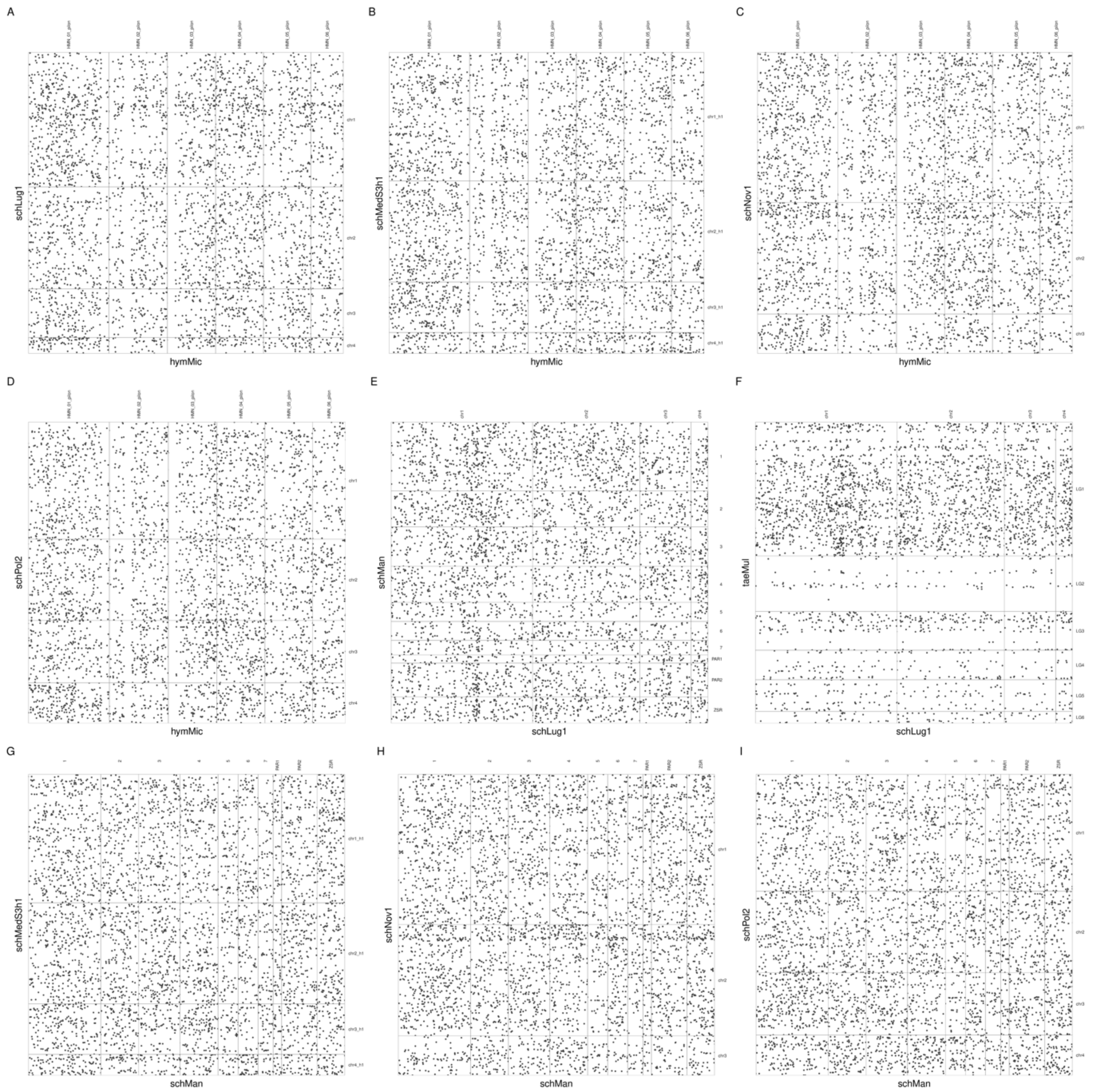

Figure 17 Oxford dotplots between the *Schmidtea* and parasites species included in this study. A *Hymenolepis microstoma* vs *S. lugubris*. B *Hymenolepis microstoma* vs *S. mediterranea*. C *Hymenolepis microstoma* vs *S. nova*. D *Hymenolepis microstoma* vs *S. polychroa*. E *S. lugubris* vs *Schistosoma mansoni*. F *S. lugubris* vs *Taenia multiceps*. G *Schistosoma mansoni* vs *S. mediterranea*. H *Schistosoma mansoni* vs *S. nova*. I *Schistosoma mansoni* vs *S. polychroa*.

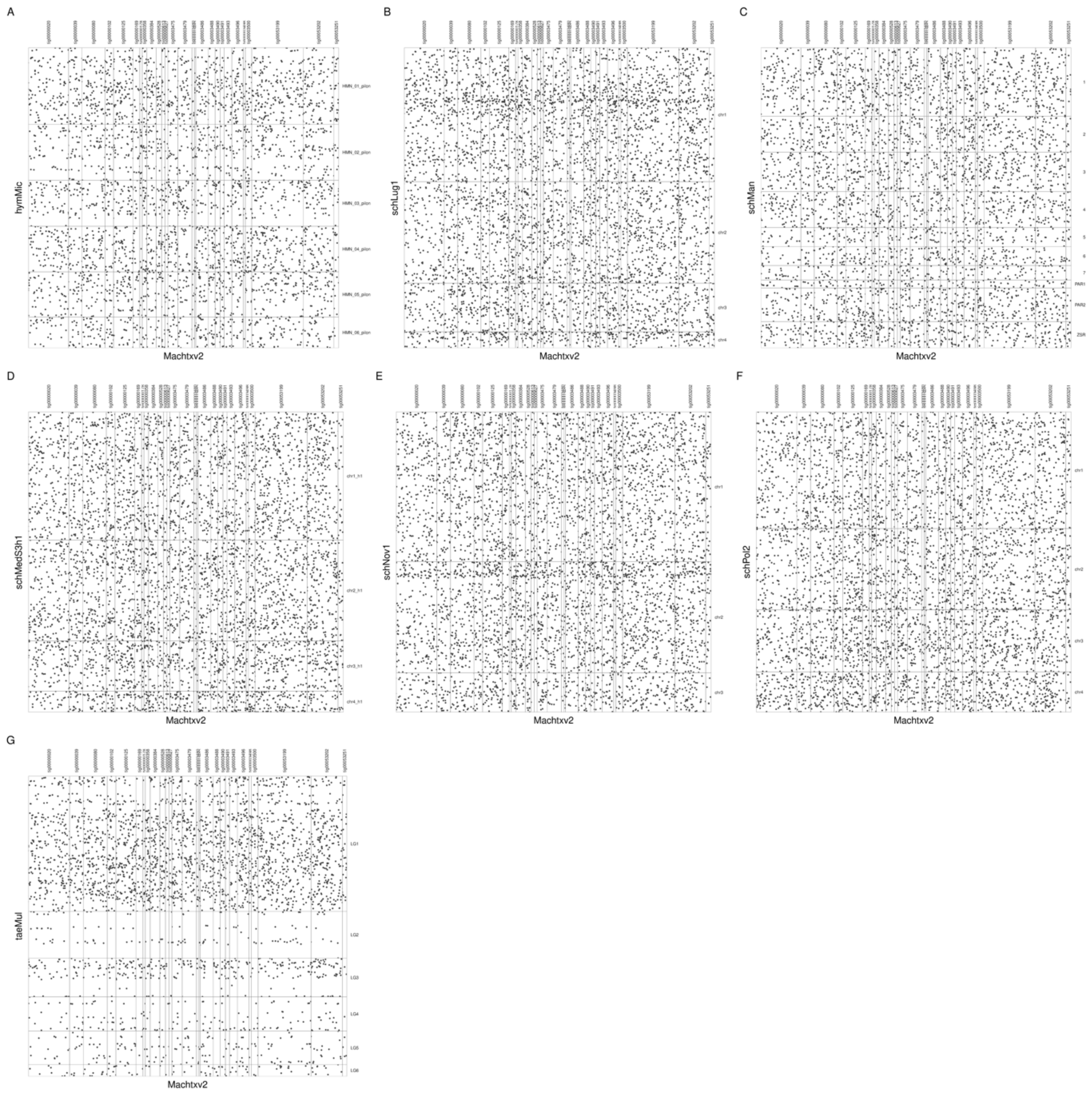

Figure 18 Oxford dotplots between *Macrostomum hystrix* and the *Schmidtea* and parasite species included in this study. A *Macrostomum hystrix* vs *Hymenolepis microstoma*. B *Macrostomum hystrix* vs *S. lugubris*. C *Macrostomum hystrix* vs *Schistosoma mansoni*. D *Macrostomum hystrix* vs *S. mediterranea*. E *Macrostomum hystrix* vs *S. nova*. F *Macrostomum hystrix* vs *S. polychroa*. G *Macrostomum hystrix* vs *Taenia multiceps*.

4.5 MALG conservation

Here we describe the conservation of the Metazoan ancestral linkage groups (MALG) defined by [5]. Following their notation, we use ⊗ to denote the fusion and mixing of two MALG and describe the equivalence of chromosomes. Note, that none of the MALG were conserved in *S. mediterranea*, *S. polychroa*, *S. nova, S. lugubris* (Figure 17A-D), or *Macrostomum hystrix* (Figure 14I) and therefore they are not included in this description. Table 15 gives the conserved MALGs for each chromosome based on the ODP results visualized in Figure 17E-H.

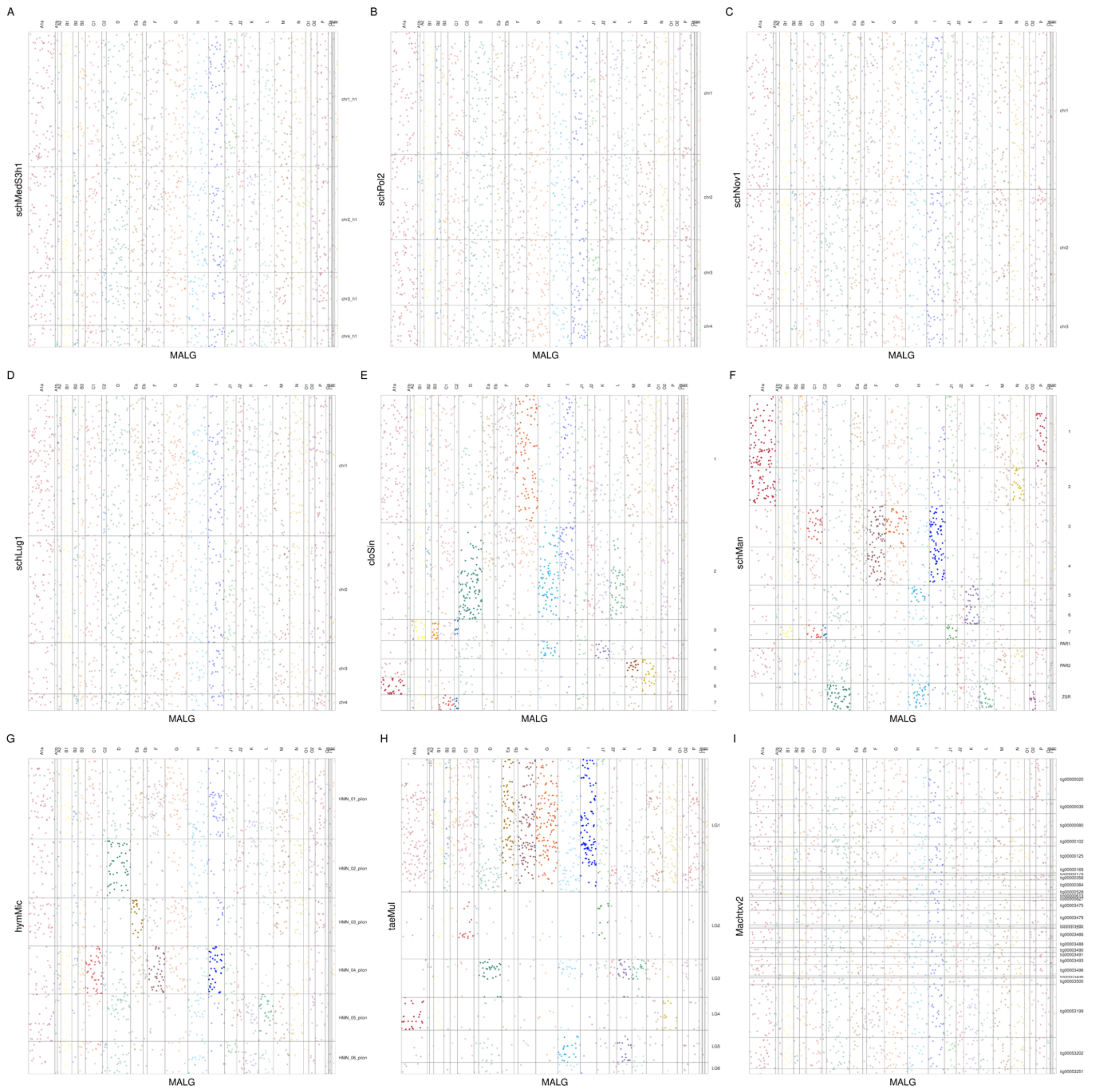

Figure 19 Oxford dotplots between MALG and the species in this study. A *S. mediterranea* haplotype 1. B *S. polychroa*. C *S. nova*. D *S. lugubris*. E *Clonorchis sinensis*. F *Schistosoma mansoni*. G *Hymenolepis microstoma*. H *Taenia multiceps*. I *Macrostomum hystrix*.

Table 15 Description of conservation of metazoan Ancestral Linkage Groups in four parasitic flatworms. Fusion and mixing of Ancestral Linkage Groups are denoted by ⊗.

species	scaffold	MALG	scaffold name
schMan	1	A1a ⊗ P	SM_V9_1
schMan	2	A1a ⊗ N	SM_V9_2
schMan	3	G ⊗ F ⊗ I ⊗ C1	SM_V9_3
schMan	4	F ⊗ I	SM_V9_4
schMan	5	H ⊗ K	SM_V9_5
schMan	6	K	SM_V9_6
schMan	7	C1 ⊗ C2 ⊗ J1	SM_V9_7
schMan	PAR1	—	SM_V9_PAR1
schMan	PAR2	—	SM_V9_PAR2
schMan	WSR	—	SM_V9_WSR
schMan	ZSR	D ⊗ H ⊗ L ⊗ O	SM_V9_ZSR
cloSin	1	G ⊗ F ⊗ I ⊗ C1	1
cloSin	2	D ⊗ H ⊗ L	2
cloSin	3	B1 ⊗ B3 ⊗ C2	3
cloSin	4	H ⊗ K	4
cloSin	5	M ⊗ N	5
cloSin	6	A1a ⊗ N	6
cloSin	7	C1 ⊗ C2	7
taeMul	1	Ea ⊗ Eb ⊗ G ⊗ F ⊗ I	LG1
taeMul	2	C1 ⊗ C2 ⊗ J1	LG2
taeMul	3	D ⊗ K ⊗ L	LG3
taeMul	4	A1a ⊗ N	LG4
taeMul	5	H ⊗ K	LG5
taeMul	6	—	LG6
hymMic	1	—	HMN_01_pilon
hymMic	2	D	HMN_02_pilon
hymMic	3	Ea	HMN_03_pilon
hymMic	4	F ⊗ I ⊗ C1	HMN_04_pilon
hymMic	5	L	HMN_05_pilon
hymMic	6	—	HMN_06_pilon

5 Genome annotations

Part I. Transcriptome assembly pipeline

This section describes the genome annotation pipeline developed for the *de novo* annotation of all *Schmidtea* genomes reported in this study. In general, it comprises an evidence-based (i.e. based on RNA sequencing data), genome-guided approach, which is summarised in the following workflow. A detailed block diagram of the pipeline is included at the end of Part I.

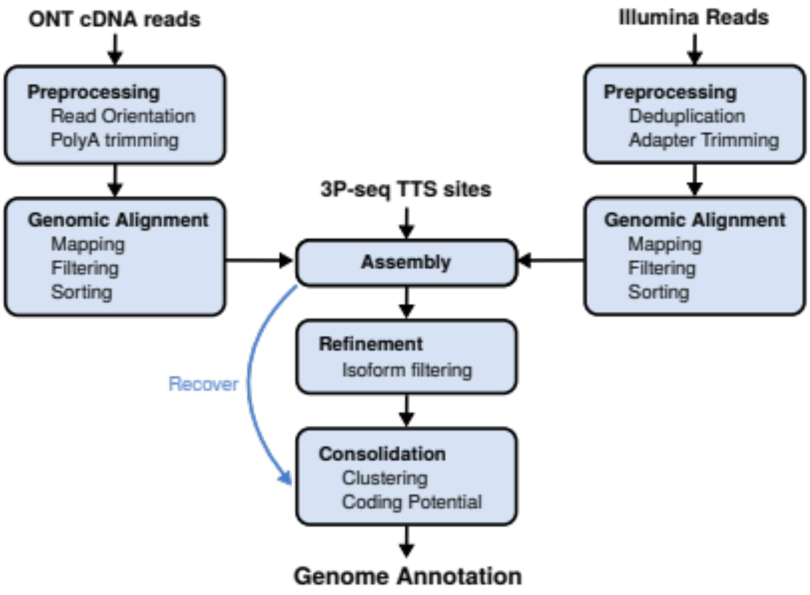

Overall workflow

5.1 Nanopore read pre-processing

5.1.1 Basecalling

ONT reads are obtained by basecalling `fast5` files using **guppy** *(6.2.1)*:

```
guppy_basecaller -i $RUNID/fast5/ \
                 -s $RUNID/fastq/ \
                 --recursive --disable_pings \
                 --trim_strategy none \
                 --compress_fastq \
                 --config $MODEL \
                 --device "cuda:0"
```

The following state-of-the art models are used for basecalling:

- *Super High Accuracy* mode for cDNA reads.

  `MODEL="dna_r9.4.1_450bps_sup_prom.cfg"`

- *High Accuracy* mode for direct RNA reads.

  `MODEL="rna_r9.4.1_70bps_hac_prom.cfg"`

Only the *pass* reads of each run (*i.e.* Q-score>10 for cDNA and >7 for direct RNA) are retained and merged into a single `fastq` file.

```
find $RUNID/fastq/pass/ -type f -name *.fastq.gz |\
xargs cat > $RUNID".sup.fq.gz"
```

5.1.2 Read orientation

Raw ONT reads are oriented and adapter-trimmed according to their native strandedness using **pychopper** *(v2.7.1)* :

```
pychopper -k PCS111 -r $RUNID"_classifier_report.pdf" -t $THREADS \
          $RUNID".sup.fq.gz" $RUNID"_flr.pre.fq"
```

This step is skipped for *direct RNA* reads, that are inherently stranded. In the previous step, as well as throughout this workflow, `$THREADS` indicates the number of available CPU threads.

5.1.3 Read trimming and filtering

Oriented reads > 150 nt are retained, then poly-A tails are trimmed and low complexity reads (arising from sequencing artifacts) are removed using **BBMap** *(38.87)* tools.

```
reformat.sh in=$RUNID.filt_flr.pre.fq out=stdout.fq minlength=150 qin=33 |\
bbduk.sh -Xmx50G in=stdin.fq out=stdout.fq trimpolya=5
         threads=$THREADS qin=33 int=f |\
bbduk.sh -Xmx50G in=stdin.fq out=$RUNID.filt_flr_nopolya.fq.gz entropy=0.2 \
         entropywindow=25 entropytrim=r threads=$THREADS qin=33 int=f
```

Finally, multiple runs are pooled into a single long read file:

```
cat *.fq.gz > $SPECIES.filt_flr_nopolya.fq.gz
```

5.1.4 Genomic alignment

Reads are aligned to the genome *fasta* using **minimap2** *(2.24)* and *single mapping* reads are saved in `bam` format. A maximum intron length of 100kb is allowed during mapping.

```
minimap2 -ax splice -G 100k -L -uf -t $THREADS \
          $SPECIES.genome.fa $SPECIES.filt_flr_nopolya.fq.gz |\
samtools view -b -F 2308 -o $SPECIES"_np.pre.bam"
```

While strongly ameliorated by the latest library preparation strategies, it is still possible that a fraction of the mapping reads arise from an internal priming event (*i.e.* oligo-dT cDNA primer annealing to an A-rich sequence within the transcript). Such events are discarded by filtering according to the genomic content around the 3’ end of the read using **seqkit** *(v0.15.0)*:

```
seqkit bam -j $THREADS \
           -x -T 'AlnContext: {
                  Ref: $SPECIES.genome.fa,
                  LeftShift: -24,
                  RightShift: 24,
                  RegexEnd: "[Aa]{8,}",
                  Stranded: True,
                  Invert: True }' $SPECIES"_np.pre.bam" > $SPECIES"_np.bam"
```

Finally, alignment files are sorted and indexed with **samtools** *(1.16.1)*:

```
samtools sort -@ $THREADS -o $SPECIES"_np.sort.bam" $SPECIES"_np.bam"
samtools index  $SPECIES"_np.sort.bam"
```

5.2 Illumina short read pre-processing

5.2.1 Adapter removal and quality trimming

Illumina Adapters and low quality regions are trimmed away from short reads using **BBduk** (from BBMap tools):

```
bbduk.sh in1=$RUNID"_R1.fq.gz" in2=$RUNID"_R2.fq.gz" \
         out1=$RUNID"_tmp_R1.fq.gz" out2=$RUNID"_tmp_R2.fq.gz" \
         ref=adapters ktrim=r k=23 mink=11 hdist=1 qtrim=rl \
         trimq=10 tpe tbo &> $RUNID.trim.log
```

5.2.2 PCR duplicate removal

In order to maximise the information content of short reads and avoid redundant mappings, we remove putative PCR duplicates (*i.e.* identical reads) using **BBmap**.

```
clumpify.sh in1=$RUNID"_tmp_R1.fq.gz" in2=$RUNID"_tmp_R2.fq.gz" \
            out1=$RUNID"_clean_R1.fq.gz" out2=$RUNID"_clean_R2.fq.gz" \
            dedupe=t optical=f &> $RUNID.clump.log
```

5.2.3 Read length filtering

FInally, reads > 40bp were filtered using **BBMap**:

```
reformat.sh in1=$RUNID"_R1.fq.gz" in2=$RUNID"_R2.fq.gz" \
            out1=$RUNID"_final_R1.fq.gz" out2=$RUNID"_final_R2.fq.gz" \
            minlength=40
```

In the case of single read libraries, the `in1|in2` and `out1|out2` arguments from paragraphs 2.1-2.3 are replaced with `in=` and `out=`, respectively.

5.2.4 Read merging by library type

Most of the datasets include:

- `ISR` (Inward paired-end, stranded:first-strand)
- `SR` (Single read, stranded:first-strand)
- `IU` (Inward paired-end, unstranded)
- `U` (Single read, unstranded)

Runs of the same type were merged into a single `fastq` file (for paired-end runs `_R1` and `_R2` are kept separated).

```
cd $SPECIES

# IU
cat IU/*R1.fq.gz > IU_R1.fq.gz
cat IU/*R2.fq.gz > IU_R2.fq.gz

# ISR
cat ISR/*R1.fq.gz > ISR_R1.fq.gz
cat ISR/*R2.fq.gz > ISR_R2.fq.gz

# U
cat U/*.fq.gz > U.fq.gz

# SR
cat SR/*.fq.gz > SR.fq.gz
```

S. lugubris, *S. polychroa* and *S. nova* are assembled using `ISR` reads only.

5.2.5 Short read genome mapping

Short reads are mapped to the genome using **HISAT2** *(2.2.1)*, after generating indexes:

```
hisat2-build -p $THREADS $SPECIES.genome.fa $SPECIES.HISAT2_index
```

Genomic alignment is performed by allowing a maximum intron length of 100kb (identical to long reads), and only single mapping reads are retained in the final `bam` files.

```
cd $SPECIES

## ISR
hisat2 -x $SPECIES.HISAT2_index -1 ISR_final_R1.fq.gz -2 ISR_final_R2.fq.gz \
       --rna-strandness RF --max-intronlen 100000 --threads $THREADS | \
       samtools view -h -f 0x2 | \
       grep -P "^\@|NH:i:1$" | \
       samtools view -h -b -o ISR_final.$SPECIES.bam

## IU
hisat2 -x $SPECIES.HISAT2_index -1 IU_final_R1.fq.gz -2 IU_final_R2.fq.gz \
       --max-intronlen 100000 --threads $THREADS | \
       samtools view -h -f 0x2 | \
       grep -P "^\@|NH:i:1$" |
       samtools view -h -b -o IU_final.$SPECIES.bam

## SR
hisat2 -x $SPECIES.HISAT2_index -U SR_final.fq.gz --rna-strandness RF \
       --max-intronlen 100000 --threads $THREADS | \
       grep -P "^\@|NH:i:1$" | \
       samtools view -h -b -o SR_final.$SPECIES.bam

## U
hisat2 -x $SPECIES.HISAT2_index -U U_final.fq.gz \
       --max-intronlen 100000 --threads $THREADS | \
       grep -P "^\@|NH:i:1$" | \
       samtools view -h -b -o U_final.$SPECIES.bam
```

Finally, alignment files from all library types of the same species are merged into a single file, sorted and indexed:

```
samtools merge -@ $THREADS  All_sreads.$SPECIES.bam \
                            SR_final.$SPECIES.bam ISR_final.$SPECIES.bam \
                            IU_final.$SPECIES.bam U_final.$SPECIES.bam

samtools sort -@ $THREADS -o All_sreads_sorted.$SPECIES.bam \
              All_sreads.$SPECIES.bam

samtools index All_sreads_sorted.$SPECIES.bam
```

5.3 Preparation of RNA Poly-adenylation site data

Poly(A)-position profiling data for S. mediterranea (*Lakshmanan et al., 2016*; SRP070102) were reanalysed using the publicly available pipeline:

https://github.com/VairavanL/3PSeq_analysis

It is important to notice that this pipeline strictly requires the input fast files to be uncompressed and to have a `.fastq` extension (*i.e.* `.fq` or other suffixes are not accepted). Furthermore, if the chromosome names contain underscore symbols, the resulting `3P-seq_processed_filtered.bedcount` output files will be formatted incorrectly (the program replaces the `_` with a `TAB`), therefore it is necessary to restore the right format using `sed`, as shown below. Briefly, short reads are mapped to the genome using **Bowtie** *(1.3.1)*, and a series of Python scripts extract the 3'-end positions. Only sites with at least 3 supporting reads are considered a valid TSS.

```
# Pre-process short reads
./3Pseq_iniprocess.py -q 3P-seq.fastq

# Prepare Bowtie index
bowtie-build --threads $THREADS $SPECIES.genome.fa $SPECIES.genome

# Align reads
./alignment_trigger.py -q 3P-seq_processed.fastq \
                       -g $SPECIES.genome -c config.ini

# Fix the formatting issues (in this case, for haplotype h1):
sed 's/\th1/_h1/g' 3P-seq_processed_filtered.bedcount |\
sed 's/\tscaffold\t/_scaffold_/g' > 3P-seq_processed_filtered.$SPECIES.bedcount

# Collect read signal into peaks
./detect_peaks.py 3P-seq_processed_filtered.$SPECIES.bedcount 3 \
> "TTS_"$SPECIES.bed
```

Finally, a custom Rscript formats the `.bed` TTS file into a point-feature (.ptf) for subsequent analysis:

```
Rscript ptfFromBed.R "TTS_"$SPECIES.bed  # generates "TTS_"$SPECIES.ptf
```

5.4 Transcriptome assembly

5.4.1 Draft transcriptome construction

Draft genomic annotations are generated **Stringtie2** *(2.2.0)* using alignments derived either from:

- ONT long-read only
- ONT long + Illumina short reads

The former approach is better suited for reconstructing an overall robust (albeit slightly less sensitive) exon chaining, the latter for fine-grained gene structures at the expenses of a slightly higher proportion of artifacts (chimaeras, spurious transcripts, etc.). The threshold parameters have been fine-tuned to optimise the sensitivity versus specificity of the assembly. Both steps incorporate Poly(A)-position by 3P-seq in the form of .ptf data.

```
# ONT long-read only
stringtie ONT/$SPECIES"_np.sort.bam" -v \
        --rf \
        -t -c 1.5 -f 0.02 -g 0 \
        -p $THREADS -m 200 -l N \
        --ptf "TTS_"$SPECIES.ptf \
        -o $SPECIES.NP.gtf

# ONT long + Illumina short reads
stringtie -v \
  --mix All_sreads_sorted.$SPECIES.bam $SPECIES"_np.sort.bam" \
  --rf \
  -c 1.5 -f 0.05 \
  -s 2.5 -g 0 \
  -p $THREADS \
  -m 200 \
  -l H \
  --ptf "TTS_"$SPECIES.ptf \
  -o $SPECIES.mix_ann.gtf
```

The output derived by the two Stringtie2 branches is combined into a low-confidence draft set of annotations `.mix_ann-V5M` :

```
stringtie --merge \
    -G $SPECIES.mix_ann.gtf \
    $SPECIES.NP.gtf \
    -o $SPECIES.mix_ann-V5M.gtf \
    -p $THREADS -F 0 -T 0 -f 0 -g 0
```

5.4.2 Model refinement

The low confidence set of annotation is refined using **FLAIR** *(v1.5)*: this program is able to remove spurious antisenses, to trim chimaeras, refine the splice junctions and filter out potential artifacts retaining transcripts with a higher confidence. All these steps are performed by relying on ONT read evidence only:

```
# Create input bed12 files from alignments
python bam2Bed12.py -i $SPECIES"_np.sort.bam" > $SPECIES"_np.sort.bed12"

# Correct reads
flair correct -q $SPECIES"_np.sort.bed12" \
              -g $SPECIES.genome.fa \
              -f $SPECIES.mix_ann-V5M.gtf \
              -o $SPECIES \
              -t $THREADS

# Collapse reads and refine loci
flair collapse -g $SPECIES.genome.fa \
               -r $SPECIES.filt_flr_nopolya.fq.gz \
               -q $SPECIES"_all_corrected.bed" \
               -f $SPECIES.mix_ann-V5M.gtf \
               -o $SPECIES.mix_ann-V5f \
               -t $THREADS \
               --temp_dir /tmp/
```

FLAIR produces a higher confidence transcript set called `.mix_ann-V5M.gtf`

5.4.3 Transcript recovery

FLAIR ONT-based refinement of the transcriptome is sometimes too harsh, resulting in cases of gene fragmentation and overall decrease in completeness. Therefore, a recovery strategy is enforced: if a locus was able to produce a longer protein before FLAIR filtering (roughly 10-15% of the cases), we include the transcript corresponding to that ORF (*i.e.* a transcript contained in `.mix_ann-V5M.gtf`) back into the high-confidence annotation. In order to do so, we first need to obtain the coding products of the two transcript collections, using the `getORF.sh` wrapper script, built around **TransDecoder** *(v5.5.0)*:

```
getORFs.sh $SPECIES.mix_ann-V5M.gtf $SPECIES.genome.fa
getORFs.sh $SPECIES.mix_ann-V5f.isoforms.gtf $SPECIES.genome.fa
```

The script produces a `GFF3` annotation (`.CDS.gff3`), a list of *gene:transcript* name pairs (`.names`), and two `fasta` files containing the nucleotide (`.CDS.cds`) and amino acid (`.CDS.pep`) sequences, respectively, of the predicted Open Reading Frames > 100 amino acids. Then we have to rename the FLAIR output transcripts (`V5f`) according to the nomenclature in `V5M` using **gffcompare** *(0.12.6)*:

```
gffcompare -o $SPECIES.MatchMissingID \
           -r $SPECIES.mix_ann-V5M.gtf \
           $SPECIES.mix_ann-V5f.isoforms.gtf
```

Finally, we perform transcript recovery based on coding potential using the custom Rscript `fragmDetectFix.R`. This script:

- Imports both pre- and post-FLAIR annotations and ORF predictions, recovering only FLAIR's `chr:XXXXX` transcripts that map completely internally to a `V5M` transcript;
- Compares side by side the predicted protein products;
- Finds which transcripts have been fragmented after FLAIR refinement;
- Inserts back transcripts with longest ORF from `V5M` .

```
Rscript fragmDetectFix.R $SPECIES.mix_ann-V5M.gtf \
                         $SPECIES.mix_ann-V5M.CDS.gff3 \
                         $SPECIES.mix_ann-V5M.names \
                         $SPECIES.mix_ann-V5f.isoforms.gtf \
                         $SPECIES.mix_ann-V5f.isoforms.CDS.gff3
                         $SPECIES.mix_ann-V5f.isoforms.names \
                         $SPECIES.MatchMissingID.$SPECIES.mix_ann-V5f.isoforms.gtf.tmap \
                         $SPECIES.mix_ann-V5MF22.gtf
```

The result is the curated transcriptome annotation file `V5MF22.gtf` .

5.4.4 Final transcript polishing

Some transcript at this stage still represent subportions of longer, full-length mRNAs. These are removed by collapsing them:

```
gffread -o $SPECIES.mix_ann-V5MF22C.gff3 \
        --merge -K -Y $SPECIES.mix_ann-V5MF22.gtf
```

Then, transcript are clustered into loci and renamed by genomic position into *gene-like* groups, using a custom script `tidyUpRename.sh` built as a wrapper around `gffread` :

```
tidyUpRename.sh $SPECIES.mix_ann-V5MF22C.gff3 \
                $SPECIES.mix_ann-V5MF22CN.gtf $TXROOT
```

`$TXROOT` represents the intended suffix of transcript names (eg. `h1Smed`, `Slug`).

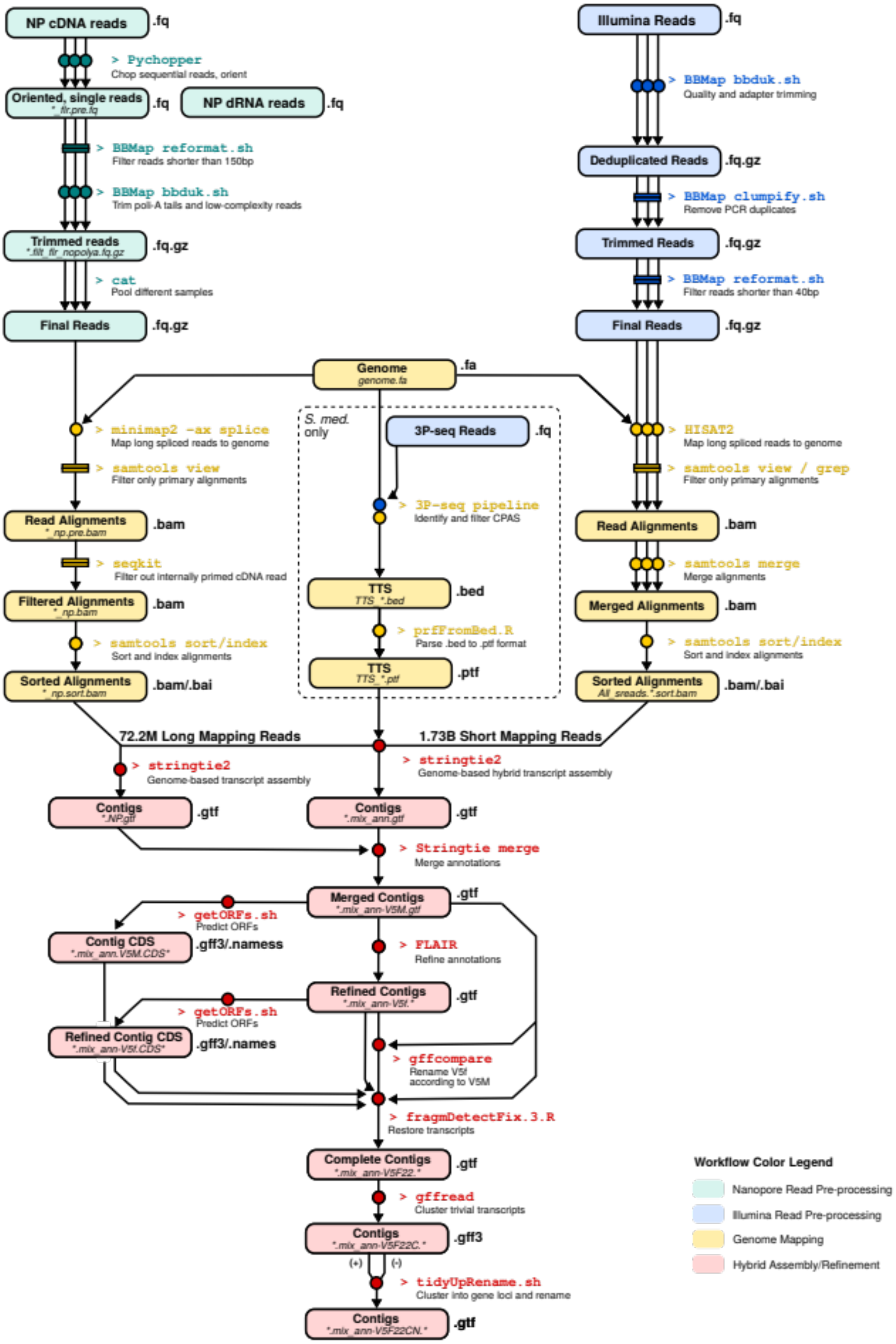

Detailed workflow of Part I

Part II. High-confidence transcript filtering

At this stage, the transcriptome is composed of a broad set of genes, each of which encompasses a collection of different isoforms. While many of these can represent true biological variants, a fraction of these is still made up by artifactual transcripts. We then proceed to filter out the obvious byproducts (e.g. intron retention events, transcript fragments, unlikely exon boundaries) in order to have a more succinct, representative snapshot of the transcriptome.

5.5 Isoform filtering

We first perform a "soft" isoform filtering, *i.e.* we reduce the isoform number by removing artifactual splicing variants but retaining the ones with good protein coding potential support. In order to proceed, we first obtain the coding potential of the transcriptome:

```
getORFs.sh $SPECIES.mix_ann-V5MF22CN.gtf $SPECIES.fa
```

Then we use the custom script `filterIsoORF.R` built as a wrapper around **CD-HIT** *(v4.8.1)* that performs the following tasks:

- Import the genomic annotations and ORF content predictions;
- For each transcript:
  - Consider only its longest ORF and drop the other shorter putative ones;
- For each gene:
  - Run ORF (protein) clustering with CD-HIT (90% identity threshold);
  - Retain the top 2 transcripts for CD-HIT each cluster with the longest ORF, allowing ties;
  - In case of ties (>1 tx with identical ORF length), keep the 2 tx with shortest tx length (this is a conservative approach, since longer products may represent chimaeras or retained introns);
- Recover genes for which no ORF was predicted for any of its transcripts and divert them into a separate file (in order to preserve very short monoexonic genes or ncRNAs).

The script it is invoked with:

```
Rscript scripts/filterIsoORF.R \
               $SPECIES.mix_ann-V5MF22CN.gtf \
               $SPECIES.mix_ann-V5MF22CN.CDS.pep \
               $SPECIES.mix_ann-V5MF22CN_FiltIso.gtf \
               $SPECIES.mix_ann-V5MF22CN_nc.gtf \
               /path/to/cd-hit-binaries/
```

It produces:

- a filtered coding isoform file `V5MF22CN_FiltIso.gtf`
- a putative ncRNA file `V5MF22CN_nc.gtf` .

After this step, both files require a further round of locus/transcript renaming and clustering. Before running `tidyUpRename.sh` this time we need to parse these `.GTF` files into valid `GFF3` format (since `gffread` does not like the formatting produced by the `rtracklayer` library used in the R script):

```
# Do an intermediate gtf->gff3 conversion
gffread -o $SPECIES.mix_ann-V5MF22CN_FiltIso.gff3 \
           $SPECIES.mix_ann-V5MF22CN_FiltIso.gtf

gffread -o $SPECIES.mix_ann-V5MF22CN_nc.gff3 \
           $SPECIES.mix_ann-V5MF22CN_nc.gtf

# Tidy up and rename gene loci and transcripts
./scripts/tidyUpRename.sh $SPECIES.mix_ann-V5MF22CN_FiltIso.gff3 \
                          $SPECIES.mix_ann-V5MF22CN_FiltIsoN.gtf
                          $SPECIES"c"

./scripts/tidyUpRename.sh $SPECIES.mix_ann-V5MF22CN_nc.gff3 \
                          $SPECIES.mix_ann-V5MF22CN_ncN.gtf
                          $SPECIES"n"
```

This time, as a last argument, we specify with the suffix “c” or “n” whether the transcript belongs to the coding or non-coding set, respectively (e.g. `h1Smedc`, `Slugn`).

5.6 Chimaeric transcript filtering

While this hybrid transcriptome assembly approach results in a lower proportion of transcript fusions, with the subsequent step we attempt to fix different kinds of remaining errors:

- **Chimaeric Loci** *i.e.* Bundle of physically different, but overlapping transcripts that are mistakenly assigned to the same Gene. This is a common event.
- **Chimaeric Transcripts** Transcripts that encode two distinct ORFs but are assembled as a single fusion transcript.
- **Mix of both**

The script `ChimaeraDetect.R` works as follow:

- For each locus:
  - Create genomic ranges with the `min` and `max` coordinates of all the predicted open reading frames;
  - Reduce these coordinates by locus, creating *range_blocks* ;
  - If the locus has 0 or 1 range_block, this is a normal locus and it can be discarded from further analysis;
- For each chimaeric locus:
  - Map each range_block(s) to its overlapping transcript(s);

– Transcripts encompassing more than one rangeblock are putative chimaeras;

– If we manage to represent all range_blocks using non-chimeric transcript, we tag the chimeric transcript(s) from the locus as redundant. Otherwise, we keep it/them and flag it/them. In this way, in the case a locus harbors a chimeric transcript *as well as* their correct counterparts, only the redundant chimaera is removed. Conversely, in the rare event that the locus only contains a chimaera, and/or the removal of this transcript would result in the loss of protein coding information, the transcript is preserved and tagged for future curation.

- Remove redundant chimaeric transcripts and rename loci accordingly (e.g. splitting chimeric loci into distinct ORF units);

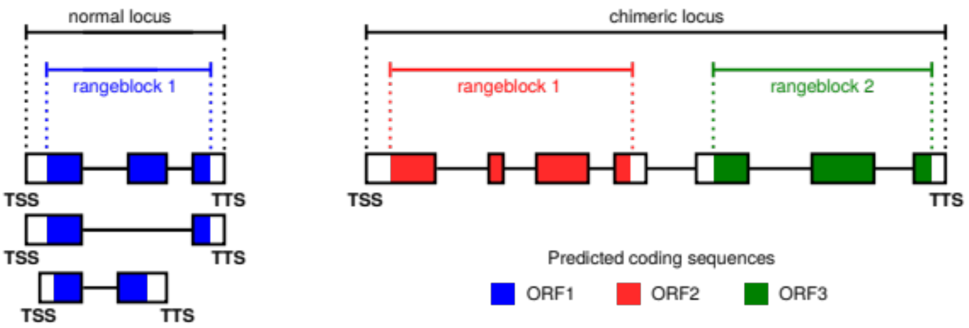

Normal and Chimaeric locus

ORF predictions are run before and after this step in order to identify and output the coding regions within the transcripts:

```
# The chimaera filtering steps require intermediate files,
# normally discarded after ORF predictions.
# With the "keeptmp" flag we retain them
./scripts/getORFs.sh $SPECIES.mix_ann-V5MF22CN_FiltIsoN.gtf \
                     $SPECIES.fa keeptmp

## Chimaera Filtering
Rscript scripts/ChimaeraDetect.R $SPECIES.mix_ann-V5MF22CN_FiltIsoN.gtf \
                                 $SPECIES.mix_ann-V5MF22CN_FiltIsoN.CDS.gff3 \
                                 $SPECIES.mix_ann-V5MF22CN_FiltIsoN.rawCDS.gff3 \
                                 $SPECIES.mix_ann-V5MF22CN_FiltIsoN_chfx.gtf \
                                 $SPECIES"c"

# Coding predictions after chimaera removal
./scripts/getORFs.sh $SPECIES.mix_ann-V5MF22CN_FiltIsoN_chfx.gtf \
                     $SPECIES.fa
```

The script returns the `V5MF22CN_FiltIsoN_chfx.gtf` file containing an amended coding transcript annotation.

5.7 Fix partial 5' ORF annotations

The ORF annotation predictions returned by TransDecoder classify the putative peptide as `complete`, `5-prime partial`, `3-prime partial`, or `internal`. However, most of the `5-prime partial` sequences contain a methionine relatively close to the predicted start of the

ORF, and manual inspection (e.g. using BLAST on the full-length protein) confirmed that many of these were actual `complete` ORFs misassigned as partial fragments. In order to fix this problem we applied the following strategy:

- Import ORF annotations
- Calculate the 5' UTR length distribution for `complete` ORFs
- Estimate a threshold for 5' UTR length by fitting a gaussian mixture model assuming a bimodal distribution with a majority of real, complete ORFs and a smaller, artifactual proportion of misassignments: the longest allowed lenght is therefore defined as 3 standard deviations above the mean of the lower population, as shown in the following picture;

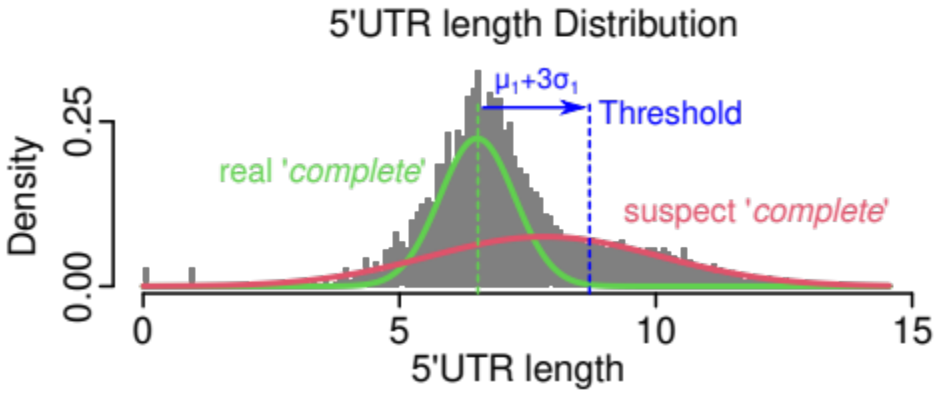

- `5-prime partial` ORFs with an in-frame methionine in their 5' UTR falling within the threshold distance are reclassified as `complete`, and their starting sites are amended accordingly.

The above approach is implemented by the script `Fix5ORF.R`:

```
Rscript scripts/Fix5ORF.R $SPECIES.mix_ann-V5MF22CN_FiltIsoN_chfx.CDS.gff3 \
                          $SPECIES.fa \
                          $SPECIES.mix_ann-V5MF22CN_FiltIsoN_chfx.fix5utr.gff3
```

The script returns a `V5MF22CN_FiltIsoN_chfx.fix5utr.gff3` file containing an amended ORF prediction annotation.

In order to produce a compact ORF set, since TransDecoder may return multiple coding sequences per transcript, we finally run the script `filter_longestORFperTx.R`:

```
# Keep only 1 ORF (the longest) per tx
Rscript scripts/filter_longestORFperTx.R \
                $SPECIES.mix_ann-V5MF22CN_FiltIsoN_chfx.fix5utr.gff3 \
                $SPECIES.mix_ann-V5MF22CN_FiltIsoN_chfx.fix5utr.uniqueCDS.gff3

# Remove the ORF number (e.g. ".p1") in place
sed -i -E 's/\.p[0-9]+//g' \
    $SPECIES.mix_ann-V5MF22CN_FiltIsoN_chfx.fix5utr.uniqueCDS.gff3
```

5.8 Final formatting

As a final step, we want to merge the coding and non-coding annotations into a single set:

```
# Convert non-coding set to gff3
gffread -F --keep-exon-attrs $SPECIES.mix_ann-V5MF22CN_ncN.gtf > \
                             $SPECIES.mix_ann-V5MF22CN_ncN.gff3

# Merge it with coding set
cat $SPECIES.mix_ann-V5MF22CN_FiltIsoN_chfx.fix5utr.uniqueCDS.gff3 \
    $SPECIES.mix_ann-V5MF22CN_ncN.gff3 |\
grep -v "#" > $SPECIES.ENCODE_hybrid_annot.tmp.gff3
```

The resulting `$SPECIES.ENCODE_hybrid_annot.tmp.gff3` annotation is then parsed using the `IDpatch.R` script. This command polishes the `GFF3` file by resolving format inconsistencies (e.g. adds explicit `gene` entries, renames the file `source`, fixes the `type` field layout):

```
Rscript scripts/IDpatch.R $SPECIES.ENCODE_hybrid_annot.tmp.gff3 \
                          $SPECIES.ENCODE_hybrid_annot.gff3
```

The following figure represents a description the overall naming scheme used in this annotation:

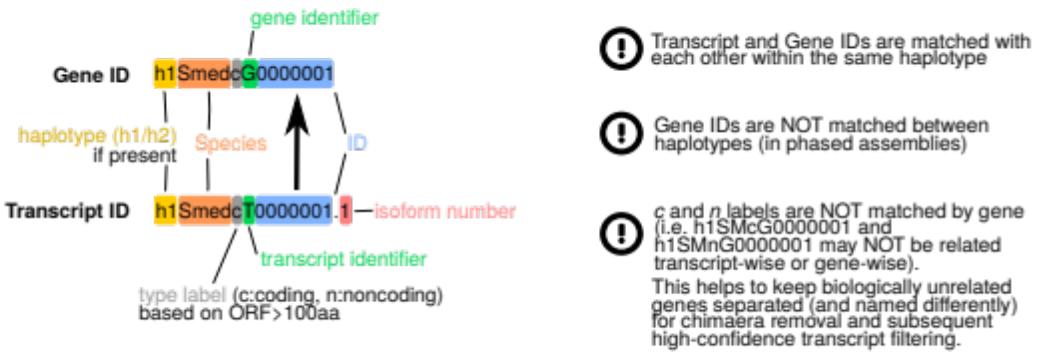

Gene naming scheme

In conclusion the file is sorted:

```
gffread --sort-alpha -F --keep-exon-attrs --keep-genes \
        $SPECIES.ENCODE_hybrid_annot.gff3 |\
grep -v "#" > $SPECIES.ENCODE_hybrid_annot.sort.gff3
```

This results in the final annotation file `ENCODE_hybrid_annot.sort.gff3` .

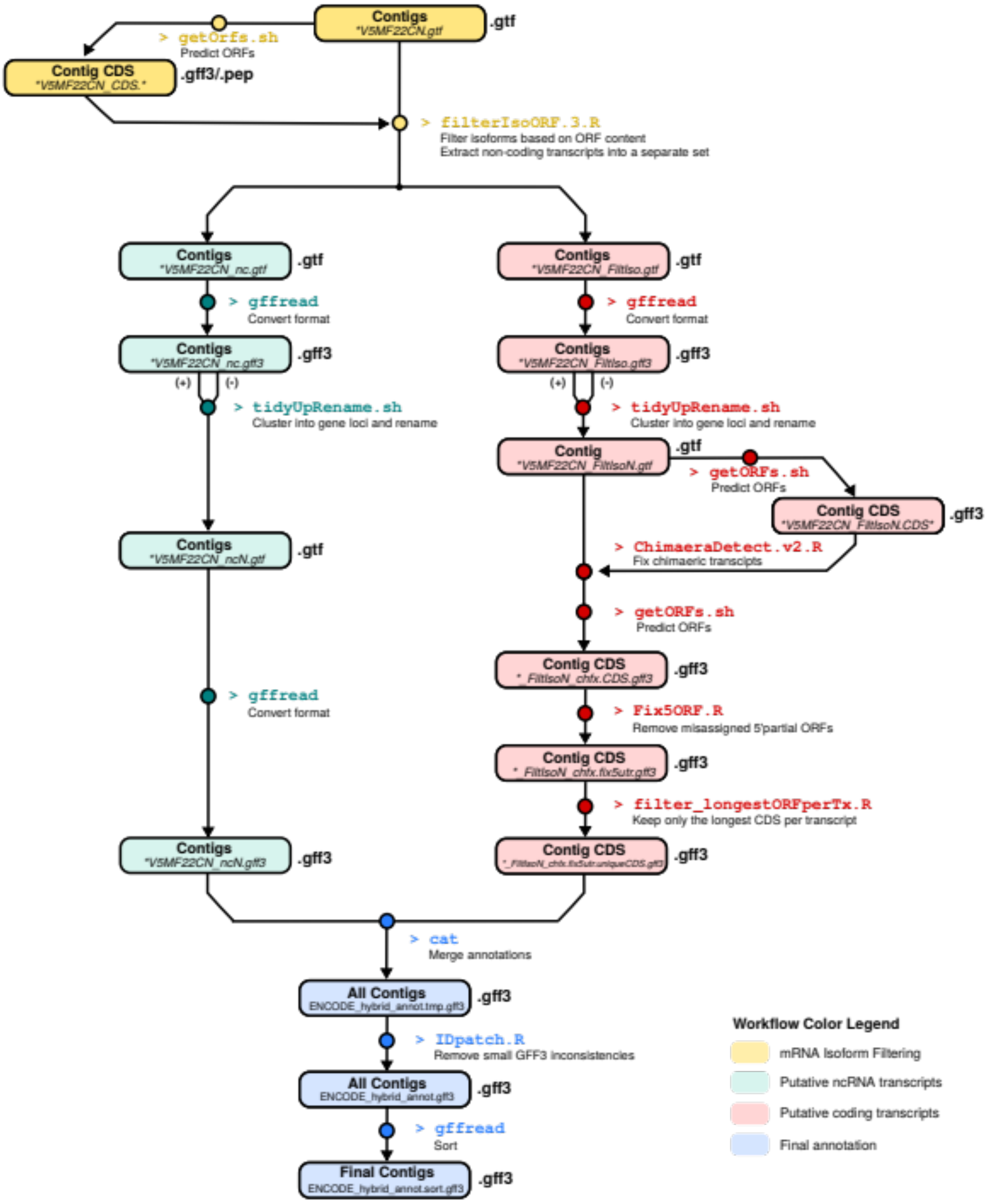

Detailed workflow of Part II

6 References

1. Grohme MA, Schloissnig S, Rozanski A, Pippel M, Young GR, Winkler S, et al. The genome of *Schmidtea mediterranea* and the evolution of core cellular mechanisms. Nature. 2018;554:56–61.

2. Guo L, Bloom JS, Dols-Serrate D, Boocock J, Ben-David E, Schubert OT, et al. Island-specific evolution of a sex-primed autosome in a sexual planarian. Nature. 2022;606:329–34.

3. An Y, Kawaguchi A, Zhao C, Toyoda A, Sharifi-Zarchi A, Mousavi SA, et al. Draft genome of Dugesia japonica provides insights into conserved regulatory elements of the brain restriction gene nou-darake in planarians. Zoological Letters. 2018;4:24.

4. Simakov O, Marletaz F, Cho S-J, Edsinger-Gonzales E, Havlak P, Hellsten U, et al. Insights into bilaterian evolution from three spiralian genomes. Nature. 2013;493:526–31.

5. Simakov O, Bredeson J, Berkoff K, Marletaz F, Mitros T, Schultz DT, et al. Deeply conserved synteny and the evolution of metazoan chromosomes. Science Advances. 2022;8:eabi5884.
