# Supplementary material for: A comparative analysis of planarian genomes reveals regulatory conservation in the face of rapid structural divergence
Source: Nat Commun. 2024 Sep 19;15:8215. doi: 10.1038/s41467-024-52380-9 (PMC11410931; doi:10.1038/s41467-024-52380-9)
Supplement: Supplementary file 3 — Description Of Additional Supplementary Files [file 41467_2024_52380_MOESM3_ESM.pdf]

Description of Additional supplementary files

File Name: Supplementary Data 1

Description: ATAC-seq peaks called using MACS2, in *Schmidtea mediterranea* assembly schMedS3h1. P-values are based on one-sided tests. Q-values give p-values adjusted for multiple comparisons using the Benjamini–Hochberg procedure. Given is the conservation scoring, putative regulatory element type derived from the intersection with ChIP-seq data, gene annotation, peak calling statistics, and orthology of the associated gene. When peaks are conserved, then the corresponding peaks and associated genes in the other Schmidtea species are indicated in the last columns. The last column shows if the associated genes in all species share the same orthogroup.

File Name: Supplementary Data 2

Description: All called H3K4me3 ChIP-seq peaks in *Schmidtea mediterranea* assembly schMedS3h1. P-values are based on one-sided tests. Q-values give p-values adjusted for multiple comparisons using the Benjamini–Hochberg procedure.

File Name: Supplementary Data 3

Description: All called H3K27ac ChIP-seq peaks in *Schmidtea mediterranea* assembly schMedS3h1. P-values are based on one-sided tests. Q-values give p-values adjusted for multiple comparisons using the Benjamini–Hochberg procedure.

File Name: Supplementary Data 4

Description: Transcriptome summary statistics of all *Schmidtea* assemblies.

File Name: Supplementary Data 5

Description: List of all metazoan BUSCO genes detected in all transcriptomes used in this study.

File Name: Supplementary Data 6

Description: Names of the genes identified by BUSCO for each *Schmidtea* transcriptome used in this study.

File Name: Supplementary Data 7

Description: All called ATAC-seq peaks in *Schmidtea polychroa* assembly schPol2. Given is the gene annotation, peak calling statistics, and orthogroup of the associated gene. P-values are based on one-sided tests. Q-values give p-values adjusted for multiple comparisons using the Benjamini–Hochberg procedure.

File Name: Supplementary Data 8

Description: All called ATAC-seq peaks in *Schmidtea nova* assembly schNov1. Given is the gene annotation, peak calling statistics, and orthogroup of the associated gene. P-values are based on one-sided tests. Q-values give p-values adjusted for multiple comparisons using the Benjamini–Hochberg procedure.

File Name: Supplementary Data 9

Description: All called ATAC-seq peaks in *Schmidtea lugubris* assembly schLug1. Given is the gene annotation, peak calling statistics, and orthogroup of the associated gene. P-values are based on one-sided tests. Q-values give p-values adjusted for multiple comparisons using the Benjamini–Hochberg procedure.

File Name: Supplementary Data 10

Description: Results of alignment-based liftover of all called *Schmidtea mediterranea* ATAC-seq peaks (Supplementary Data 1) onto *Schmidtea polychroa* assembly schPol2. Indicated are the liftover coordinates, the *S. mediterranea* peak name, if the *S. mediterranea* summit was lifted over, and if the liftover overlaps with a *S. polychroa* ATAC-seq peak.

File Name: Supplementary Data 11

Description: Results of alignment-based liftover of all called *Schmidtea mediterranea* ATAC-seq peaks (Supplementary Data 1) onto *Schmidtea nova* assembly schNov1. Indicated are the liftover coordinates, the *S. mediterranea* peak name, if the *S. mediterranea* summit was lifted over, and if the liftover overlaps with a *S. nova* ATAC-seq peak.

File Name: Supplementary Data 12

Description: Results of alignment-based liftover of all called *Schmidtea mediterranea* ATAC-seq peaks (Supplementary Data 1) onto *Schmidtea lugubris* assembly schLug1. Indicated are the liftover coordinates, the *S. mediterranea* peak name, if the *S. mediterranea* summit was lifted over, and if the liftover overlaps with a *S. lugubris* ATAC-seq peak.

File Name: Supplementary Data 13

Description: Orthologs inferred between the four *Schmidtea* species using GENESPACE.

File Name: Supplementary Data 14

Description: Synteny blocks between the four *Schmidtea* species inferred using GENESPACE.

File Name: Supplementary Data 15

Description: Test for enrichment of repetitive elements at synteny breakpoints. Given is the observed number of elements and the mean and standard deviation of elements in each simulation of reference (ref) and target genomes. Pval_smaller and pval_larger indicate p-values for one-sided tests of the simulation from the observed value. padj_smaller and padj_larger give p-values adjusted for multiple testing using the Bonferroni method.
