# Supplementary material for: A comparative analysis of planarian genomes reveals regulatory conservation in the face of rapid structural divergence
Source: Nat Commun. 2024 Sep 19;15:8215. doi: 10.1038/s41467-024-52380-9 (PMC11410931; doi:10.1038/s41467-024-52380-9)
Supplement: Supplementary file 5 — Reporting summary [file 41467_2024_52380_MOESM5_ESM.pdf]

nature portfolio

Corresponding author(s): Jochen C. Rink

Last updated by author(s): Jul 25, 2024

Reporting Summary

Nature Portfolio wishes to improve the reproducibility of the work that we publish. This form provides structure for consistency and transparency in reporting. For further information on Nature Portfolio policies, see our Editorial Policies and the Editorial Policy Checklist.

Statistics

For all statistical analyses, confirm that the following items are present in the figure legend, table legend, main text, or Methods section.

n/a	Confirmed	
☐	☒	The exact sample size (*n*) for each experimental group/condition, given as a discrete number and unit of measurement
☒	☐	A statement on whether measurements were taken from distinct samples or whether the same sample was measured repeatedly
☐	☒	The statistical test(s) used AND whether they are one- or two-sided<br>*Only common tests should be described solely by name; describe more complex techniques in the Methods section.*
☒	☐	A description of all covariates tested
☐	☒	A description of any assumptions or corrections, such as tests of normality and adjustment for multiple comparisons
☐	☒	A full description of the statistical parameters including central tendency (e.g. means) or other basic estimates (e.g. regression coefficient) AND variation (e.g. standard deviation) or associated estimates of uncertainty (e.g. confidence intervals)
☐	☒	For null hypothesis testing, the test statistic (e.g. *F*, *t*, *r*) with confidence intervals, effect sizes, degrees of freedom and *P* value noted<br>*Give P values as exact values whenever suitable.*
☒	☐	For Bayesian analysis, information on the choice of priors and Markov chain Monte Carlo settings
☒	☐	For hierarchical and complex designs, identification of the appropriate level for tests and full reporting of outcomes
☒	☐	Estimates of effect sizes (e.g. Cohen's *d*, Pearson's *r*), indicating how they were calculated

Our web collection on statistics for biologists contains articles on many of the points above.

Software and code

Policy information about availability of computer code

Data collection	No software was used for data collection.
Data analysis	All the software used for the analyses, including version numbers are detailed in the Methods section and the following github repository: https://github.com/Jeremias-Brand/PlanarianGenomeAnalysis.

For manuscripts utilizing custom algorithms or software that are central to the research but not yet described in published literature, software must be made available to editors and reviewers. We strongly encourage code deposition in a community repository (e.g. GitHub). See the Nature Portfolio guidelines for submitting code & software for further information.

Data

Policy information about availability of data

All manuscripts must include a data availability statement. This statement should provide the following information, where applicable:

- Accession codes, unique identifiers, or web links for publicly available datasets
- A description of any restrictions on data availability
- For clinical datasets or third party data, please ensure that the statement adheres to our policy

The whole-genome, HiC, ATAC-seq, ChIP-seq, and RNA-Seq of Schmidtea mediterranea, Schmidtea polychroa, Schmidtea nova, and Schmidtea lugubris data generated in this study have been deposited in the NCBI database under accession code PRJNA1052007 [https://www.ncbi.nlm.nih.gov/bioproject/PRJNA1052007]. The repetitive element annotation of Schmidtea genomes data generated in this study have been deposited in the Zenodo database under accession code

11004547 [https://doi.org/10.5281/zenodo.11004547]. The Clonorchis sinensis genome and annotation data used in this study are available in the NCBI database under accession code PRJNA386618 [https://www.ncbi.nlm.nih.gov/bioproject/PRJNA386618]. The Schistosoma mansoni genome and annotation data used in this study are available in the NCBI database under accession code PRJEA36577 [https://www.ncbi.nlm.nih.gov/bioproject/PRJEA36577]. The Taenia multiceps genome and annotation data used in this study are available in the NCBI database under accession code PRJNA307624 [https://www.ncbi.nlm.nih.gov/bioproject/PRJNA307624]. The Hymenolepis microstoma genome and annotation data used in this study are available in the NCBI database under accession code PRJEB124 [https://www.ncbi.nlm.nih.gov/bioproject/PRJEB124]. The Macrostomum hystrix gene annotation data used in this study are available in the Zenodo database under accession code 7861770 [https://doi.org/10.5281/zenodo.7861770]. The Macrostomum hystrix genome data used in this study are available in the European Nucleotide Archive database under accession code GCA_950097015 [https://www.ebi.ac.uk/ena/browser/view/GCA_950097015].

Research involving human participants, their data, or biological material

Policy information about studies with human participants or human data. See also policy information about sex, gender (identity/presentation), and sexual orientation and race, ethnicity and racism.

Reporting on sex and gender	*Use the terms sex (biological attribute) and gender (shaped by social and cultural circumstances) carefully in order to avoid confusing both terms. Indicate if findings apply to only one sex or gender; describe whether sex and gender were considered in study design; whether sex and/or gender was determined based on self-reporting or assigned and methods used. Provide in the source data disaggregated sex and gender data, where this information has been collected, and if consent has been obtained for sharing of individual-level data; provide overall numbers in this Reporting Summary. Please state if this information has not been collected. Report sex- and gender-based analyses where performed, justify reasons for lack of sex- and gender-based analysis.*
Reporting on race, ethnicity, or other socially relevant groupings	*Please specify the socially constructed or socially relevant categorization variable(s) used in your manuscript and explain why they were used. Please note that such variables should not be used as proxies for other socially constructed/relevant variables (for example, race or ethnicity should not be used as a proxy for socioeconomic status). Provide clear definitions of the relevant terms used, how they were provided (by the participants/respondents, the researchers, or third parties), and the method(s) used to classify people into the different categories (e.g. self-report, census or administrative data, social media data, etc.) Please provide details about how you controlled for confounding variables in your analyses.*
Population characteristics	*Describe the covariate-relevant population characteristics of the human research participants (e.g. age, genotypic information, past and current diagnosis and treatment categories). If you filled out the behavioural & social sciences study design questions and have nothing to add here, write "See above."*
Recruitment	*Describe how participants were recruited. Outline any potential self-selection bias or other biases that may be present and how these are likely to impact results.*
Ethics oversight	*Identify the organization(s) that approved the study protocol.*

Note that full information on the approval of the study protocol must also be provided in the manuscript.

Field-specific reporting

Please select the one below that is the best fit for your research. If you are not sure, read the appropriate sections before making your selection.

☒ Life sciences ☐ Behavioural & social sciences ☐ Ecological, evolutionary & environmental sciences

For a reference copy of the document with all sections, see nature.com/documents/nr-reporting-summary-flat.pdf

Life sciences study design

All studies must disclose on these points even when the disclosure is negative.

Sample size	The sample size reflects the maximum number of experiments that could be performed.
Data exclusions	NONE
Replication	ATAC-seq;3 biological replicates with 3 technical replicates for both experimental conditions. ChIP-seq; one input sample and one pull-down sample for both experimental conditions. Additional ChIP-seq experiments were conducted on x-rayed animals displaying signal decrease close to known x-ray sensitive genes (data not shown here). Principal component analysis was used to confirm the clustering of technical and biological ATAC-seq replicates. Read pileups of replicates were visually inspected. Additionally, colocalization of histone marks and accessible chromatin was observed, mirroring the situation in other model organisms.
Randomization	Experimental animals were cultured in 3 separate containers for each species and fed in a staggered interval. Wt and xray animals of each biological replicate originate from the same box. Animals were allocated at random to the experimental groups.
Blinding	Blinding was not necessary for the bioinformatic analyses.

Reporting for specific materials, systems and methods

We require information from authors about some types of materials, experimental systems and methods used in many studies. Here, indicate whether each material, system or method listed is relevant to your study. If you are not sure if a list item applies to your research, read the appropriate section before selecting a response.

Materials & experimental systems

n/a	Involved in the study
☐	☒ Antibodies
☒	☐ Eukaryotic cell lines
☒	☐ Palaeontology and archaeology
☐	☒ Animals and other organisms
☒	☐ Clinical data
☒	☐ Dual use research of concern
☒	☐ Plants

Methods

n/a	Involved in the study
☐	☒ ChIP-seq
☒	☐ Flow cytometry
☒	☐ MRI-based neuroimaging

Antibodies

Antibodies used

α-H3K4me3 (milipore 07-473 Lot#3381394); α-H3K27ac (active motif #39133 Lot#16119013)

Validation

α-H3K4me3 (merckmillipore Cat.#07-473 Lot#3381394);
α-H3K27ac (activemotif Cat.#39133 Lot#16119013)

2.5 µL of α-H3K4me3 stock per pull-down (Millipore 07-473 Lot#3381394, stock concentration: not stated)
5 µL of α-H3K27ac stock per pull-down (active motif #39133 Lot#16119013, stock concentration : 1 ug/uL)
further specifications in Methods Section

α-H3K4me3;
*Immunogen: KLH-conjugated linear peptide corresponding to 10 amino acids from the N-terminal region of human Histone H3 trimethylated on lysine 4.
*Quality Assurance by supplier: Western Blotting in acid extract of HeLa cells.
*Tested applications by supplier (https://www.merckmillipore.com/DE/en/product/Anti-trimethyl-Histone-H3-Lys4-Antibody,MM_NF-07-473#anchor_TI):
Dot Blot Analysis,Chromatin Immunoprecipitation,Immunocytochemistry
α-H3K27ac;
*Immunogen: This Histone H3 acetyl Lys27 antibody was raised against a peptide including acetyl-lysine 27 of histone H3.
*Validation by supplier: Tested by Western blot on Raji cell nuclear extract
*Tested applications by supplier (https://www.activemotif.com/catalog/details/39133/histone-h3-acetyl-lys27-antibody-pab)
ChIP-Seq, ICC/IF, WB, CUT&Tag

Animals and other research organisms

Policy information about studies involving animals; ARRIVE guidelines recommended for reporting animal research, and Sex and Gender in Research

Laboratory animals

All animals used for these analyses were derived from long-term laboratory cultures. Laboratory strain of the sexual biotype of S. mediterranea (S2F18, derived from S2F2, internal ID: GOE00500). Laboratory strain of the asexual biotype of S. mediterranea (CIW4, internal ID: GOE00071). The S. nova strain (internal ID: GOE00023) was collected at 51,0717710; 13,7421400, in Dresden, Germany on 2013-04-14. The S. lugubris strain (internal ID: GOE00057) was collected at 52.942432, -1.113739 in Nottingham, UK (JCR). The S. polychroa strain (internal ID: GOE00227) was collected at 43.71249; 16.72605 near the Village of Gala, Croatia.

Wild animals

The study did not involve wild animals.

Reporting on sex

The studied animals are simultaneous hermaphrodites or asexual.

Field-collected samples

All animal were kept under standard culture conditions implemented in the species collections of the Max Planck Institute of Molecular Cell Biology and Genetics in Dresden and the Max Planck Institute for Multidisciplinary Sciences in Göttingen.

Ethics oversight

All experiments were conducted in accordance with German law and the ethical guidlines of the Max Planck Society. No special approval is required to work with flatworms.

Note that full information on the approval of the study protocol must also be provided in the manuscript.

Plants

Seed stocks

Report on the source of all seed stocks or other plant material used. If applicable, state the seed stock centre and catalogue number. If plant specimens were collected from the field, describe the collection location, date and sampling procedures.

Novel plant genotypes

Describe the methods by which all novel plant genotypes were produced. This includes those generated by transgenic approaches, gene editing, chemical/radiation-based mutagenesis and hybridization. For transgenic lines, describe the transformation method, the number of independent lines analyzed and the generation upon which experiments were performed. For gene-edited lines, describe the editor used, the endogenous sequence targeted for editing, the targeting guide RNA sequence (if applicable) and how the editor was applied.

Authentication

Describe any authentication procedures for each seed stock used or novel genotype generated. Describe any experiments used to assess the effect of a mutation and, where applicable, how potential secondary effects (e.g. second site T-DNA insertions, mosiacism, off-target gene editing) were examined.

ChIP-seq

Data deposition

☒ Confirm that both raw and final processed data have been deposited in a public database such as GEO.

☒ Confirm that you have deposited or provided access to graph files (e.g. BED files) for the called peaks.

Data access links
May remain private before publication.

https://www.ncbi.nlm.nih.gov/bioproject?term=PRJNA1052007

Files in database submission

HJK7JBGXB_1_H3K4me3_19s002810-1-1_Ivankovic_lane119s002810_R1.fastq: wt_H3K4me3
HJK7JBGXB_1_Input_19s002812-1-1_Ivankovic_lane119s002812_R1.fastq: wt_H3K4me3_Input
L47661_merged_R1.fastq: wt_H3K27ac
L47669_merged_R1.fastq: wt_H3K27ac_Input

Genome browser session
(e.g. UCSC)

Final submission

Methodology

Replicates

none

Sequencing depth

30-70 million fragments

Antibodies

α-H3K4me3 (milipore 07-473 Lot#3381394); α-H3K27ac (active motif #39133 Lot#16119013)

Peak calling parameters

```
bwa mem -M -t {threads} {file} > {output}
samtools index {file}
picard MarkDuplicates INPUT={file.bam} OUTPUT={output.bam} REMOVE_DUPLICATES=True METRICS_FILE={output.log}
ASSUME_SORTED=True VALIDATION_STRINGENCY=LENIENT"
samtools view -h -b -q 20 -f 3 {file} > {output}

### wt H3K4me3 ###
# macs2 peak calling macs2 2.2.7.1
macs2 callpeak \
-t H3K4me3_IP \
-c H3K4me3_Input \
-n wt_H3K4me3 \
-f BAMPE \
--nomodel \
--bdg \
--keep-dup all \
-g 6.44e8

### wt H3K27ac ###
# macs2 peak calling macs2 2.2.7.1
macs2 callpeak \
-t wt_H3K27ac_IP \
-c wt_H3K27ac_Imput \
-n wt_H3K27ac \
-f BAMPE \
--nomodel \
--bdg \
--keep-dup all \
-g 6.44e8
```

Data quality	H3K4me3:<br>*number of peaks with -log10(qvalue) > 1.30: 18361<br>*number of peaks with fold_enrichment > 5: 13106<br>H3K27ac:<br>*number of peaks with -log10(qvalue) > 1.30: 38923<br>*number of peaks with fold_enrichment > 5: 14843
Software	bwa mem, samtools, picard, macs2, deeptools
